# Supplementary material for: Patient-derived parathyroid organoids as a tracer and drug-screening application model
Source: Stem Cell Reports. 2022 Oct 27;17(11):2518–30. doi: 10.1016/j.stemcr.2022.09.015 (PMC9669499; doi:10.1016/j.stemcr.2022.09.015)
Supplement: Document S1. Figures S1–S5 and supplemental experimental procedures [file mmc1.pdf]

Stem Cell Reports, Volume *17*

Supplemental Information

Patient-derived parathyroid organoids as a tracer and drug-screening application model

Milou E. Noltes, Luc H.J. Sondorp, Laura Kracht, Inês F. Antunes, René Wardenaar, Wendy Kelder, Annelies Kemper, Wiktor Szymanski, Wouter T. Zandee, Liesbeth Jansen, Adrienne H. Brouwers, Robert P. Coppes, and Schelto Kruijff

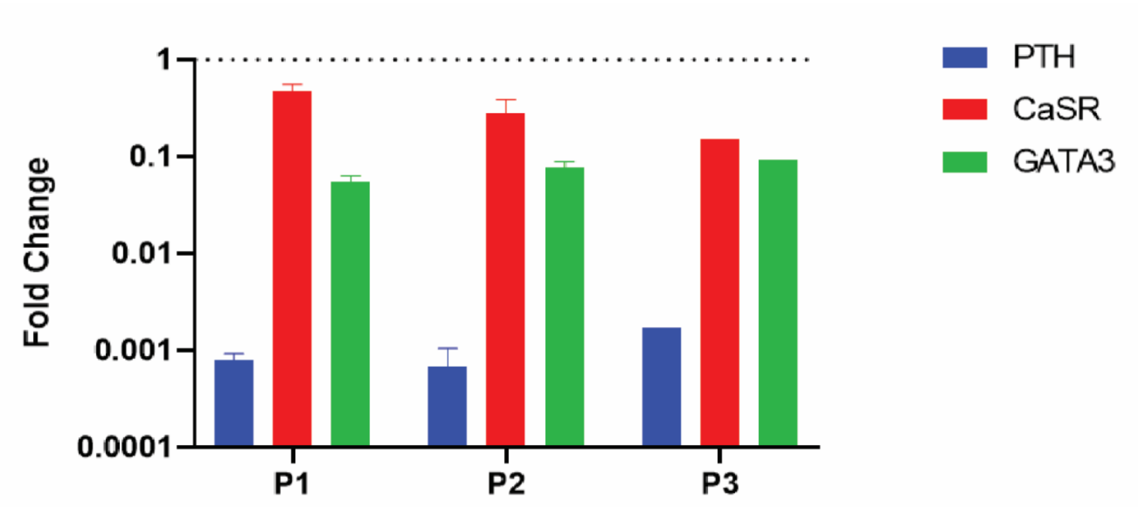

Supplemental Figure 1. Gene expression pattern analysis across multiple passages (n=3 patients for p1 and p2, n=2 for p3). Dotted line resembles tissue expression levels (n=3), and error bars resemble SEM. No significance was observed between passages with the lowest p-value being 0.065. Related to Figure 1 (self-renewal of organoid-forming cells from human putative parathyroid stem cells).

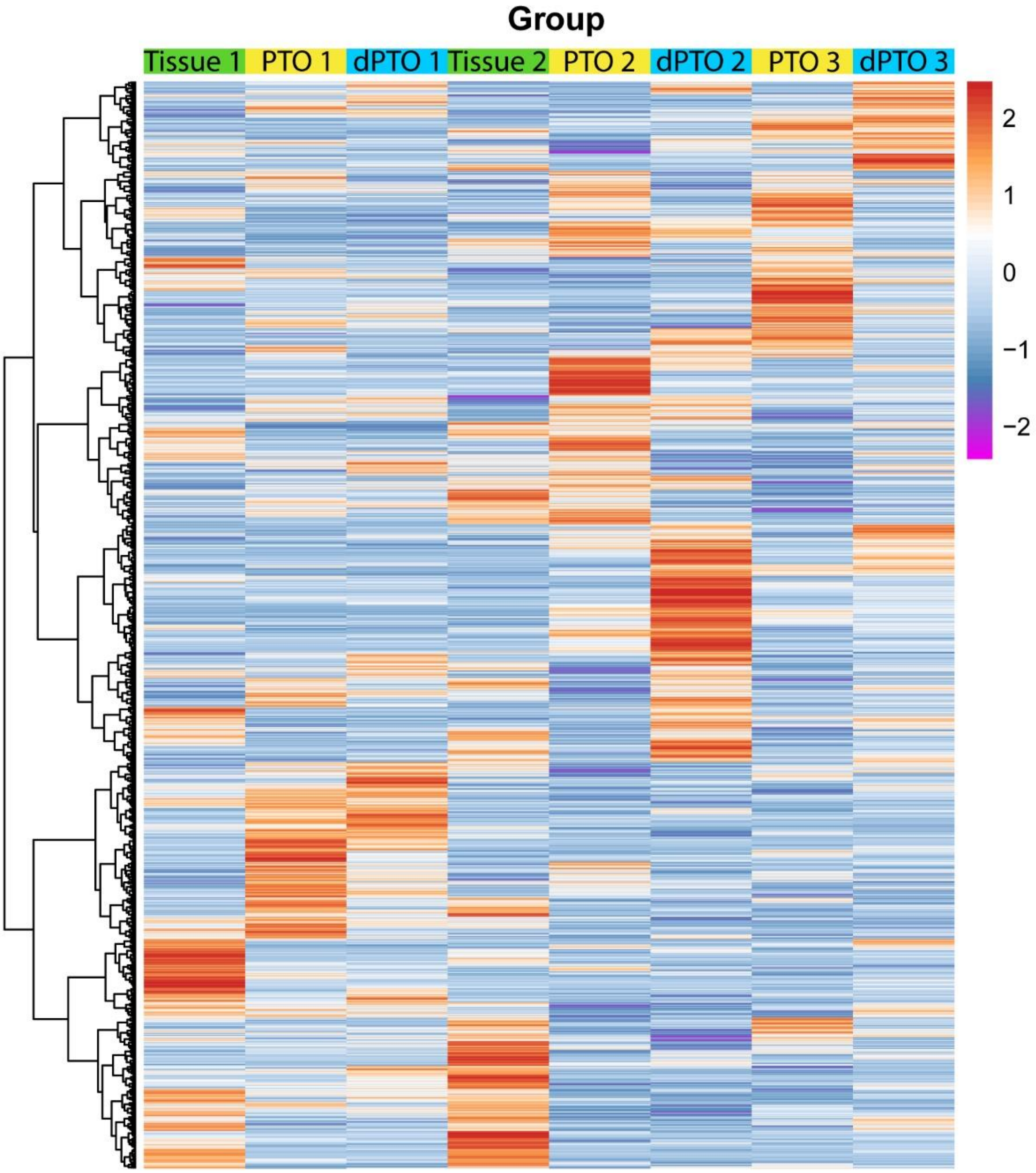

Supplemental Figure 2. Heatmap showing all sequenced genes for all included samples separately. Data is shown in row Z-score of counts per million (Supplemental table 1). *PTO*= parathyroid organoids, *dPTO*= differentiated parathyroid organoids. Related to Figure 3 (bulk RNA-sequencing).

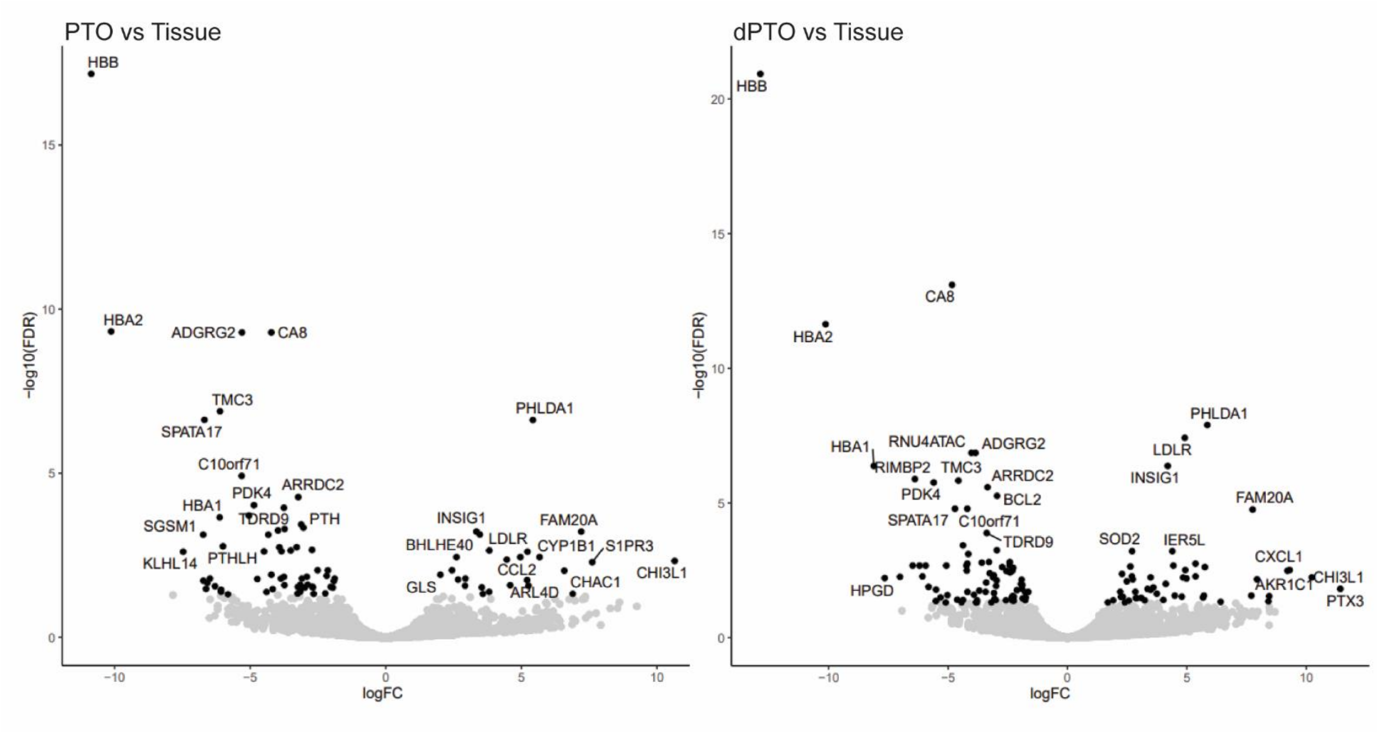

Supplemental Figure 3. Volcano plots showing differentially expressed genes (FDR<0.05) in PTO or dPTO compared to tissue (Supplemental Table 2 and 3). *PTO*= parathyroid organoids, *dPTO*= differentiated parathyroid organoids. Related to Figure 3 (bulk RNA-sequencing).

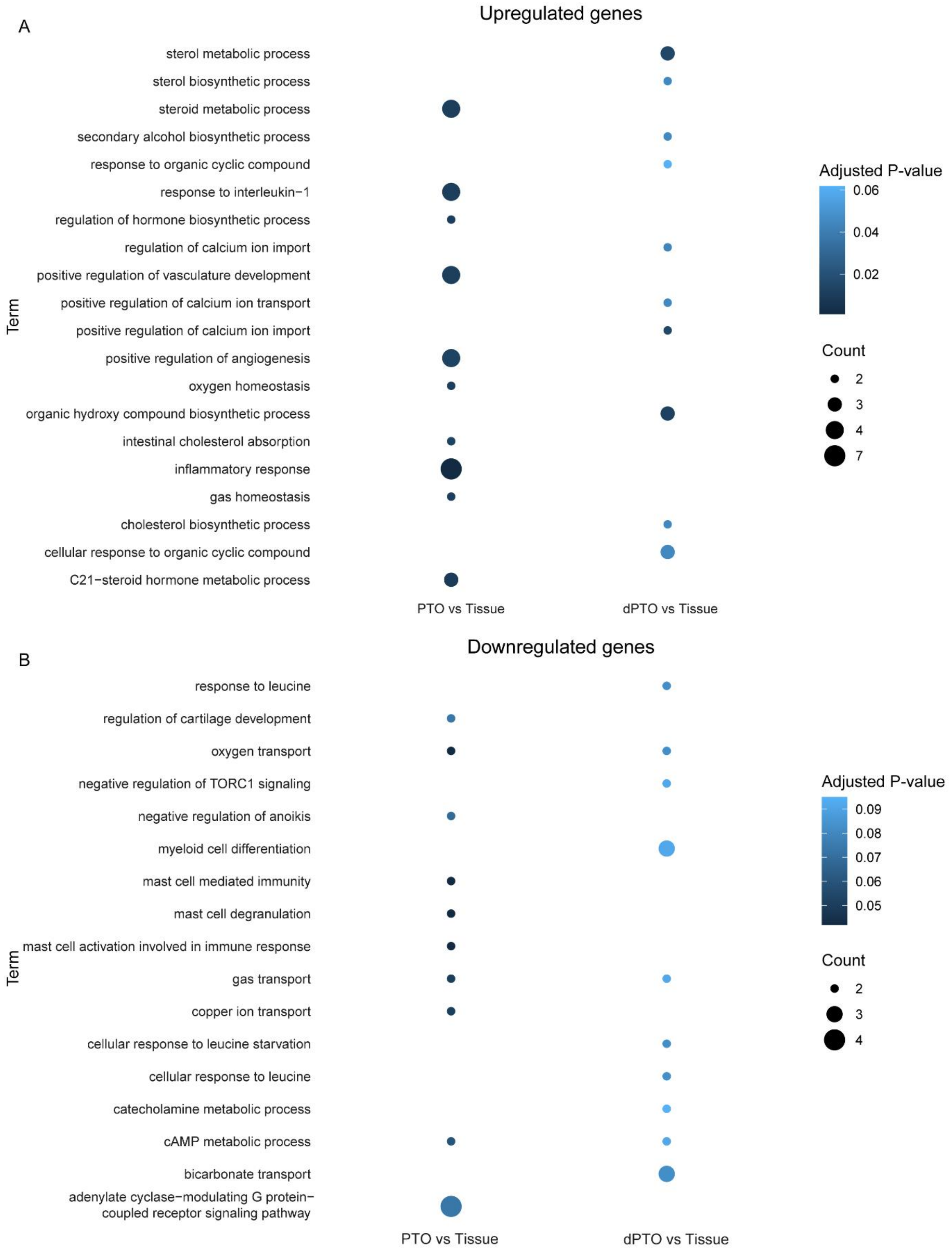

Supplemental Figure 4. Dotplots showing top ten GO terms associated with upregulated (A) and downregulated (B) genes in PTO and dPTO compared to tissue. Ranking of GO terms was based on the most significant GO terms. Dot colors resemble significance and size resembles number of associated genes *PTO*= parathyroid organoids, *dPTO*= differentiated parathyroid organoids. Related to Figure 3 (bulk RNA-sequencing).

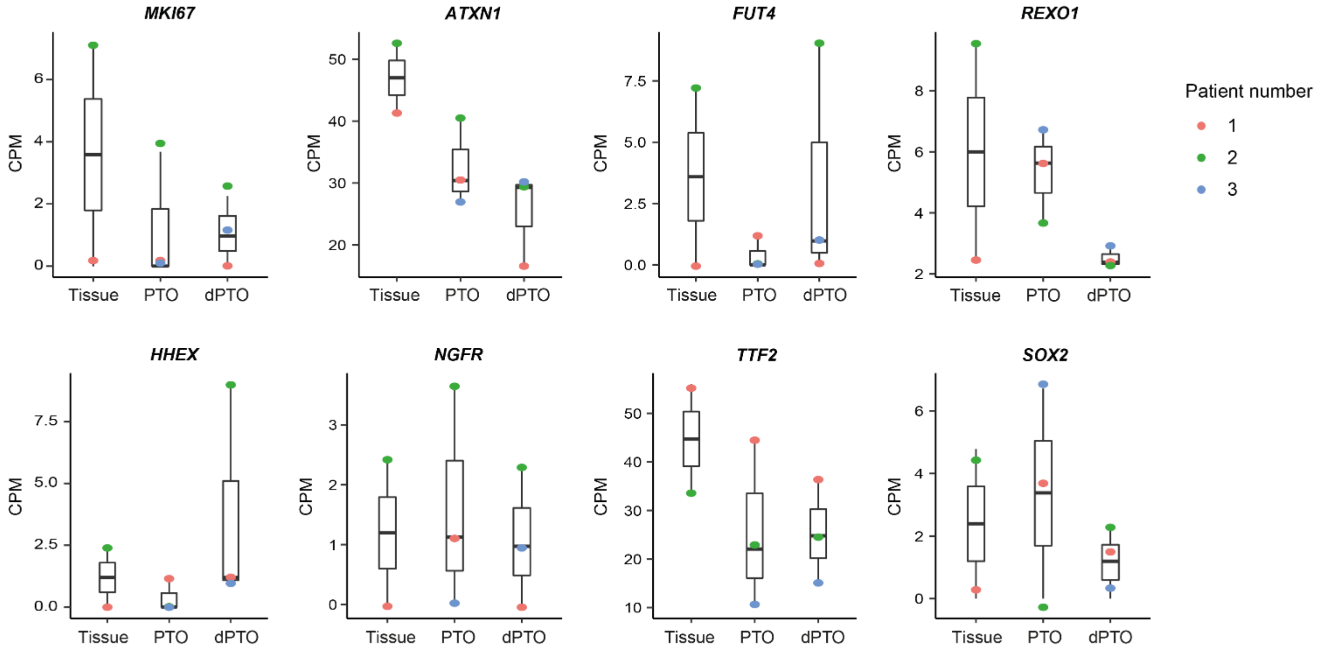

Supplemental Figure 5. Boxplots of generic stem cell markers of primary hyperplastic parathyroid tissue, PTOs at the end of passage 1 and two weeks dPTOs in counts per million (CPM). Boxplot shows median, two hinges (25th and 75th percentile) and two whiskers (largest and smallest value no further than 1.5x inter-quartile range). *PTO*= parathyroid organoids, *dPTO*= differentiated parathyroid organoids. Related to Figure 3 (bulk RNA-sequencing).

Supplemental experimental procedures

Quantitative Polymerase Chain Reaction

Total RNA from patient material (n=3) and organoids (p1 and p2 n=3, p3 n=2) was extracted (RNeasy™ Mini Kit ,Qiagen). To obtain cDNA, 500ng of total RNA was reverse transcribed using 1 µL 10 mM dNTP Mix, 100 ng random primers, 5x First-strand Buffer, 0.1 M DTT, 40 units of RNase OUT and 200 units of M-MLV RT, in a volume of 20 µL for each reaction (all Invitrogen). qPCR (Bio-Rad) was performed using Bio-Rad iQ SYBR Green Supermix according to manufacturer's instructions. For each sample, PCR buffer was mixed with 100 ng cDNA, sybergreen and forward and reverse primers for the targeted genes in a volume of 13 µL. A three-step qPCR reaction was applied. Oligo sequences of primes were as followed: PTH fwd, 5′-AGCTACTAACATACCTGAACG-3′; PTH rev, 5′-CTCTCCATCGACTTCAGATG-3′; CaSR fwd, 5′-AGATGGCACGGGACACTACC-3′; CaSR rev, 5′-AGGAGGCATAACTGACCTGGG-3′; GATA3 fwd, 5′-AAGCCTCTGCAATGTGCTC-3′; GATA3 rev, 5′-GTGGTGGTCTGACAGTTCGC-3′, and YWHAZ fwd, 5′- GATCCCCAATGCTTCACAAG-3′; YWHAZ rev, 5′- TGCTTGTTGTGACTGATCGAC -3′.
