# Supplementary material for: Patient-derived parathyroid organoids as a tracer and drug-screening application model
Source: Stem Cell Reports. 2022 Oct 27;17(11):2518–30. doi: 10.1016/j.stemcr.2022.09.015 (PMC9669499; doi:10.1016/j.stemcr.2022.09.015)
Supplement: Document S2. Article plus supplemental information [file mmc3.pdf]

Stem Cell Reports

Article

OPEN ACCESS

Patient-derived parathyroid organoids as a tracer and drug-screening application model

Milou E. Noltes,[1,2,12] Luc H.J. Sondorp,[1,3,12] Laura Kracht,[4] Inês F. Antunes,[2] René Wardenaar,[5] Wendy Kelder,[6] Annelies Kemper,[7] Wiktor Szymanski,[8,9] Wouter T. Zandee,[10] Liesbeth Jansen,[1] Adrienne H. Brouwers,[2] Robert P. Coppes,[3,11,13,*] and Schelto Kruijff[1,2,13,*]

[1]Department of Surgical Oncology, University of Groningen, University Medical Center Groningen, Groningen, the Netherlands
[2]Department of Nuclear Medicine and Molecular Imaging, University of Groningen, University Medical Center Groningen, Groningen, the Netherlands
[3]Department of Biomedical Sciences of Cells and Systems, Section Molecular Cell Biology, University of Groningen, University Medical Center Groningen, Groningen, the Netherlands
[4]Department of Biomedical Sciences of Cells and Systems, Section Molecular Neurobiology, University of Groningen, University Medical Center Groningen, Groningen, the Netherlands
[5]Department of Ageing Biology/ERIBA, University of Groningen, University Medical Center Groningen, Groningen, the Netherlands
[6]Department of Surgery, Martini Hospital, Groningen, the Netherlands
[7]Department of Surgery, Treant Hospital, Hoogeveen, the Netherlands
[8]Stratingh Institute for Chemistry, University of Groningen, Groningen, Groningen, the Netherlands
[9]Department of Radiology, University of Groningen, University Medical Center Groningen, Groningen, the Netherlands
[10]Department of Endocrinology, University of Groningen, University Medical Center Groningen, Groningen, the Netherlands
[11]Department of Radiation Oncology, University of Groningen, University Medical Center Groningen, Groningen, the Netherlands
[12]These authors contributed equally
[13]These authors contributed equally
*Correspondence: r.p.coppes@umcg.nl (R.P.C.), s.kruijff@umcg.nl (S.K.)
https://doi.org/10.1016/j.stemcr.2022.09.015

SUMMARY

Parathyroid diseases are characterized by dysregulation of calcium homeostasis and alterations in parathyroid hormone (PTH) excretion. The development of parathyroid-targeted treatment and imaging tracers could benefit from *in vitro* models. Therefore, we aim to establish a patient-derived parathyroid organoid model representing human parathyroid tissue. Hyperplastic parathyroid tissue was dispersed, and parathyroid organoids (PTOs) were cultured and characterized. PTO-derived cells exhibited self-renewal over several passages, indicative of the presence of putative stem cells. Immunofluorescence and RNA sequencing confirmed that PTOs phenocopy hyperplastic parathyroid tissue. Exposure of PTOs to increasing calcium concentrations and PTH-lowering drugs resulted in significantly reduced PTH excretion. PTOs showed specific binding of the imaging tracers ^{11}C-methionine and ^{99m}Tc-sestamibi. These data show the functionality of PTOs resembling the parathyroid. This PTO model recapitulates the originating tissue on gene and protein expression and functionality, paving the way for future physiology studies and therapeutic target and tracer discovery.

INTRODUCTION

The parathyroid glands usually are four delicate structures 3–4 mm in size, situated close to the thyroid gland, and functionally maintain calcium homeostasis by secreting parathyroid hormone (PTH). Calcium is an essential element for the nervous, muscular, and skeletal systems. Parathyroid glands consist of chief and oxyphil cells; the former express calcium-sensing receptor (CaSR) and regulate the blood calcium concentration by mediating PTH secretion (Ritter et al., 2012). Oxyphil cells are less prevalent than chief cells and increase in number with age (Christie, 1967). Parathyroid diseases are heterogeneous conditions that are generally caused by alterations in PTH secretion, resulting in dysregulation of calcium homeostasis. In primary hyperparathyroidism, reduced CaSR expression has been noted (Singh et al., 2020; Varshney et al., 2013), resulting in uncontrolled release of PTH, leading to hypercalcemia (Tfelt-Hansen and Brown, 2005).

Our understanding of parathyroid hyperplastic growth, development of effective parathyroid tracers for diagnostic imaging, and drugs for targeted treatment could benefit from *in vitro* models derived from human hyperplastic parathyroid tissue. Organoids are 3D structures that closely recapitulate tissue architecture and cellular composition and are developed from stem cells (Tuveson and Clevers, 2019). Organoid models have been developed from multiple exocrine and endocrine glandular tissues (Hu et al., 2018; Gendoo et al., 2019; McCracken et al., 2014; Ogundipe et al., 2021; Pringle et al., 2016). These models have proven very useful for studying tumor behavior and assessing drug responses and have provided a platform for long-term *in vitro* experimentation (Hu et al., 2018; Gendoo et al., 2019; McCracken et al., 2014). This study aimed to isolate human parathyroid stem cells from primary tissue and study their potential to expand and form parathyroid organoids (PTOs) *in vitro* that recapitulate functional parathyroid tissue. We assess the potential of such a patient-derived PTO model to perform physiology studies and its potential

This is an open access article under the CC BY license (http://creativecommons.org/licenses/by/4.0/).

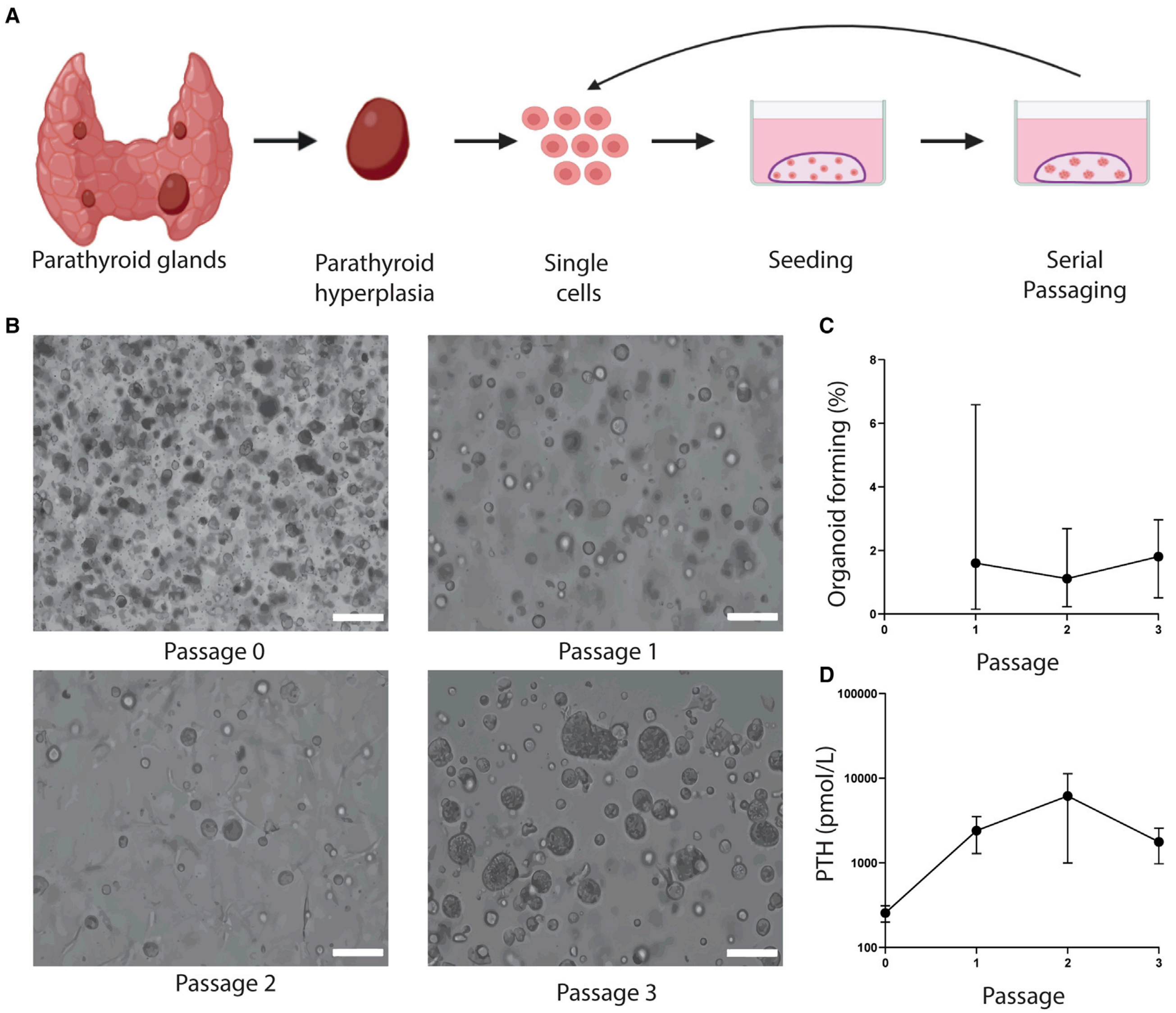

Figure 1. ***In vitro* self-renewal of organoid-forming cells from human putative parathyroid stem cells**
(A) Schematic of parathyroid tissue isolation, primary tissue culture, and the self-renewal assay.
(B) Primary parathyroid spheres 7 days in culture (p0) and PTOs at the end of p1, p2, and p3. Scale bars, 100 μm.
(C) Self-renewal potential of PTOs during passaging (n = 19 patients; error bars represent range).
(D) Secreted PTH levels from PTOs during passaging (n = 4 patients; error bars represent SEM). PTO, parathyroid organoid.
For additional information on gene expression pattern analysis across multiple passages, see Figure S1.

to be used for development of potential novel therapeutic targets, drug screening, and imaging tracer testing.

RESULTS

In vitro self-renewal and organoid formation from human parathyroid stem cells

Fifty hyperplastic parathyroid gland biopsies were dissociated into single cells and cell clumps using mechanical and enzymatic digestion. These cells were cultured and formed parathyroid spheres (Figures 1A and 1B, passage 0). To determine the presence of potential stem cells in parathyroid tissue, the *in vitro* self-renewal potential was assessed. Seventeen cultures were used to determine organoid formation efficiency (OFE), with 100% of cultures reaching passage 1 (p1), 41% reaching p2, 35% reaching p3, and 18% reaching p4 with a maximum OFE of 6.6% in p1, 2.7% in p2, and 2.9% in p3 (Figures 1B and 1C). Thus, self-renewal of parathyroid cells was shown. The PTOs

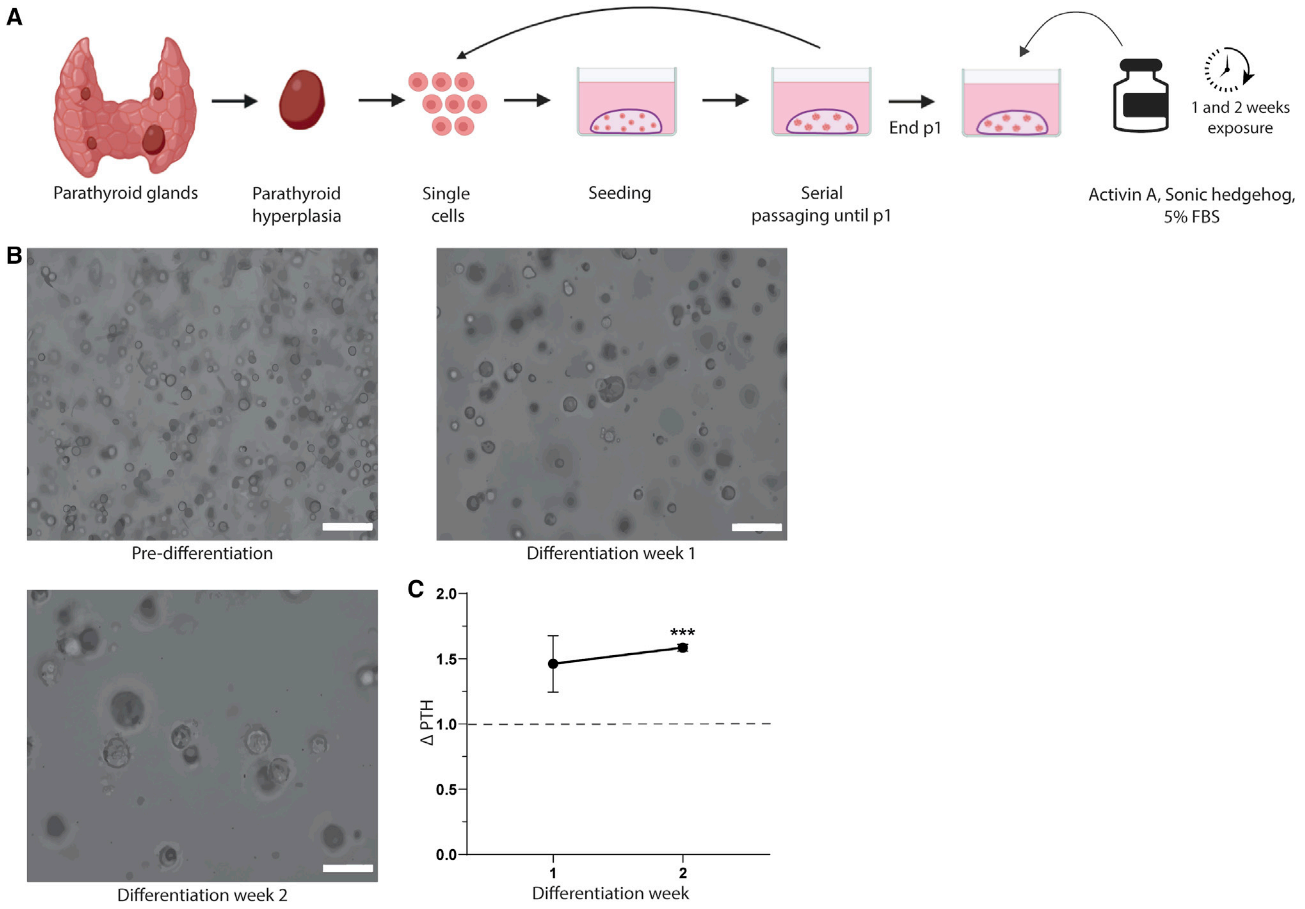

Figure 2. dPTOs

(A) Schematic of parathyroid tissue isolation, primary tissue culture, and differentiation.

(B) PTOs before differentiation (end of p1) and after 1 week and 2 weeks of differentiation. Scale bars 100 μm.

(C) Secreted PTH levels after 1 week and 2 weeks of differentiation, normalized to PTH levels prior to differentiation (n = 3 patients; error bars represent SEM). ***$p < 0.05$.

continued to secrete PTH during culture (Figure 1D). To further characterize PTOs, gene expression of parathyroid markers was evaluated by quantitative polymerase chain reaction (qPCR). Gene expression of the parathyroid markers PTH, CaSR, and GATA binding protein 3 (GATA3), a transcription factor involved in development of parathyroid tissue (Ordóñez, 2014), showed a stable expression between p1–p3 ($p > 0.065$) (Figure S1).

Characterization of PTOs

To evaluate whether the putative parathyroid stem cells are able to differentiate, the cells were subjected to a modified Bingham protocol (Bingham et al., 2009; Woods Ignatoski et al., 2010) involving continuous exposure to 100 ng/mL Activin, 100 ng/mL Sonic hedgehog (Shh), and 5% fetal bovine serum (FBS) for 21 days with medium changes every 7 days. Previous studies have shown that this protocol successfully replicates parathyroid differentiation using human embryonic stem cells or tonsil-derived mesenchymal stem cells (Bingham et al., 2009; Woods Ignatoski et al., 2010; Park et al., 2015).

During differentiation, the cells increased slightly in size, but no other morphological changes were observed (Figure 2B). PTH secretion increased during differentiation (Figure 2C). In week 2 of differentiation, the PTH levels differed significantly from the levels of PTH secreted prior to differentiation (average increase in PTH of 46.2% ± 21.6% in week 1 [$p = 0.166$] and 58.6% ± 2.6% in week 2 [$p = 0.002$]).

To further characterize PTOs and differentiated PTOs (dPTO), bulk RNA sequencing was used to compare hyperplastic parathyroid tissue (n = 2), PTOs (n = 3), and Bingham protocol dPTOs (n = 3). When assessing the differentially expressed genes (DEGs) between hyperplastic parathyroid tissue and PTOs, we only observed 25 up- and 61 downregulated genes of 14,900 evaluated genes.

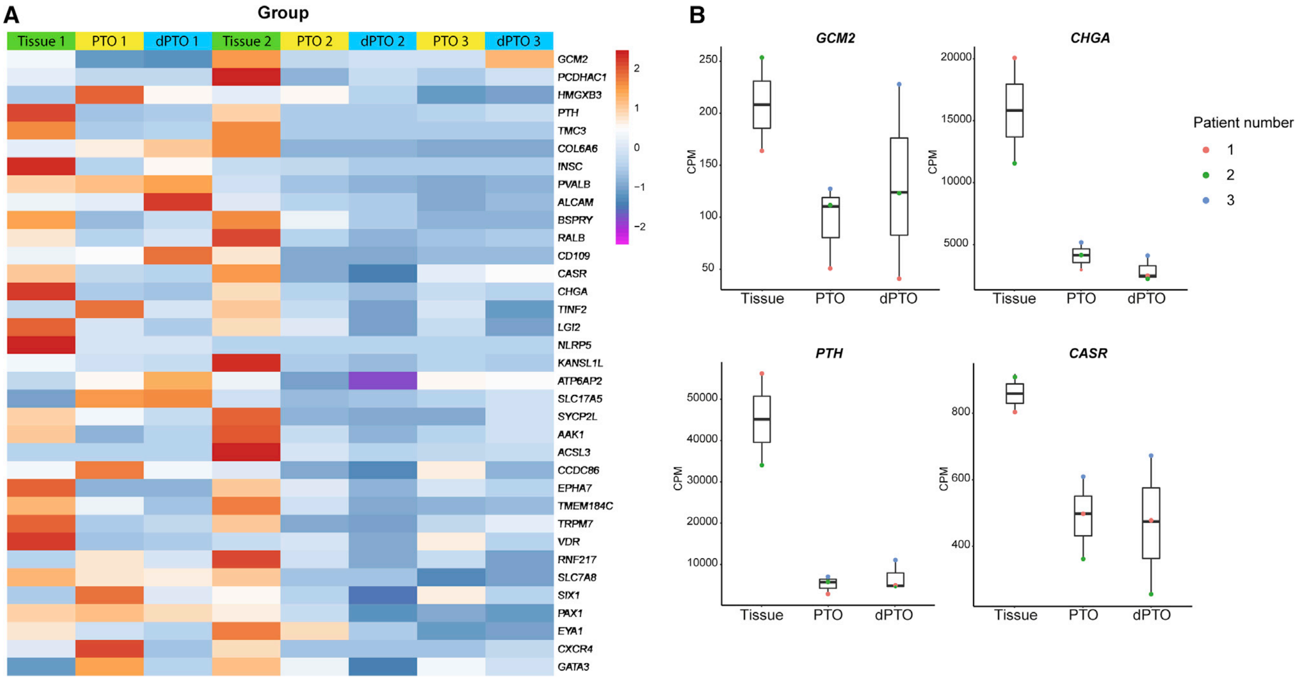

Figure 3. Bulk RNA sequencing reveals limited differences between primary hyperplastic parathyroid tissue and PTOs at the end of p1 and 2 weeks dPTOs

(A) Heatmap indicating gene expression (counts per million [CPM]) of parathyroid-specific genes in primary hyperplastic parathyroid tissue, PTOs at the end of p1, and 2-week differentiated PTOs (dPTOs).

(B) Boxplots of parathyroid-specific gene expression (CPM) in primary hyperplastic parathyroid tissue, PTOs at the end of p1, and 2-week dPTOs. The boxplot shows median, two hinges (25th and 75th percentile), and two whiskers (largest and smallest value no further than 1.5× interquartile range).

For additional information on RNA sequencing, see Figures S2–S5.

Between tissue and dPTOs, we observed 50 up- and 82 downregulated genes of 14,900 evaluated genes. Only two of 35 genes of the parathyroid markers (Figure 3) were differentially expressed in PTOs and dPTOs compared with tissue. These two genes were *PTH* and *CHGA*, both excreted factors and both downregulated in PTO and dPTOs compared with tissue. Thus, a similarity between tissue and PTOs and dPTOs was observed, especially in parathyroid-specific genes (Figure 3). However, we did observe inter-patient variability in gene expression of tissue (i.e., between tissue of patients 1 and 2), which was also reflected in PTOs and dPTOs (Figure S2; Data S1). When comparing tissue, PTOs and dPTOs of a single patient (e.g., patient 1), limited variability was observed (Figure S2).

Genes that were differentially expressed in tissue compared with PTOs and dPTOs (Figure S3; Data S2 and S3) were categorized and represented as biological processes and pathways (Gene Ontology [GO] terms; Figure S4; Data S4–S7). Genes such as *STC1* (Stanniocalcin 1) and *CCL2* (C-C motif chemokine ligand 2), which were significantly upregulated in PTOs and dPTOs compared with tissue, were among others associated with calcium ion transport (Data S4 and S6). Genes significantly upregulated in the dPTO group compared with tissue seem to be involved in regulation of cell population proliferation (Data S6). Differentiation of these organoids might be the reason of this possibly more proliferative state because, in PTOs, this GO term was not detected (Data S4 and S6). Genes significantly upregulated in PTOs compared with tissue were associated with angiogenesis and vascularization. No parathyroid-specific biological processes were associated with downregulation under PTO and dPTO conditions compared with tissue (Figure S4; Data S5 and S7).

We found that all parathyroid-specific genes (The Human Protein Atlas, 2022; Uhlén et al., 2015; Yu et al., 2015; Fagerberg et al., 2014) expressed in hyperplastic parathyroid tissue samples were also expressed in PTOs and dPTOs (Figure 3A). When we focus on four parathyroid specific genes, glial cells missing transcription factor 2 (*GCM2*), chromogranin A (*CHGA*), *PTH*, and *CASR*, gene expression of all was observed, but to a lower extent, in dPTOs and PTOs compared with primary tissue (Figure 3B). Expression of these important parathyroid markers was confirmed at the protein level (Figure 4). When comparing

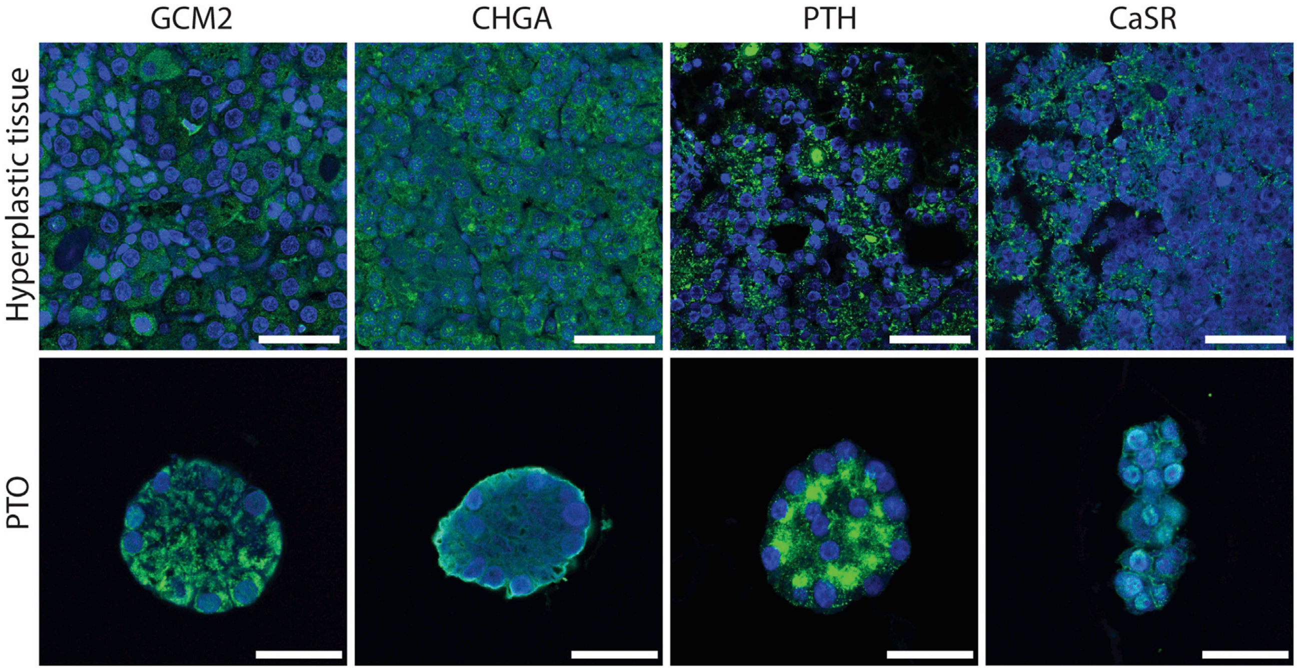

Figure 4. Immunofluorescence characterization of PTOs
Representative images of organoids and tissue, showing parathyroid-specific markers. Scale bars, 100 μm (tissue) and 25 μm (organoids). Markers indicated at the top are shown as a green fluorescent signal. Nuclei are shown as a blue fluorescent signal.

parathyroid tissue with PTOs, similarities were seen in the expression pattern of GCM2, CHGA, PTH, and CaSR (Figure 4).

To study the presence of stem cell markers in human PTOs, we analyzed the most proposed stem cell markers (van der Vaart et al., 2021) using bulk RNA-sequencing. No upregulation was observed in the putative stem cell markers when comparing PTOs and dPTOs with tissue (Figure S5). None of the stem cell markers (Figure S5) were differentially expressed in PTOs or dPTOs compared with tissue.

Functionality of PTOs

PTH secretion is negatively regulated by increased extracellular calcium levels in normal physiology. Therefore, we examined whether release of PTH by PTOs was responsive to extracellular calcium (Figure 5A). We observed a significant decrease of 31.3% ± 10.8% ($p = 0.015$) when organoids were subjected to very high levels of calcium (3.4 mM) compared with normal culture conditions (1.4 mM) (Figure 5B). When the organoids were switched back to basal 1.4 mM calcium, we observed a significant increase of 53.0% ± 11.1.% ($p = 0.018$) (Figure 5B). These results indicate a fully functional response of PTOs.

Drug screening

To demonstrate the utility of patient-derived PTOs in testing potential novel therapeutic targets, validated therapeutic agents were used to assess the effect of the drug on PTH secretion by the PTOs (Figure 6A). Cinacalcet HCl (cinacalcet), an allosteric modulator of CaSR that increases the sensitivity of the CaSR to extracellular calcium, suppresses PTH secretion in patients with hyperparathyroidism (Junaid and Patel, 2021). Calcitriol, a vitamin D analog, inhibits production of PTH by acting directly on the parathyroid gland vitamin D receptor (Hinson et al., 2010; Mannstadt, 2021). We observed a significant decrease in PTH secretion when organoids were subjected to different concentrations of cinacalcet compared with the DMSO control (average reduction of 58.9% ± 8.5% for 1.5 μM and 58.8% ± 12.9% for 2.5 μM, $p = 0.020$ and $p = 0.045$, respectively) (Figure 6B). A similar effect was observed when organoids were exposed to different concentrations of calcitriol (average reduction of 50.3% ± 3.3% for 2 nM and 54.4% ± 2.0% for 20 nM, $p < 0.0001$ and $p < 0.0001$, respectively).

Positron emission tomography (PET)/single-photon emission computed tomography (SPECT) tracer validation

To demonstrate the utility of patient-derived PTOs to validate PET/SPECT tracers, the validated tracers ^{11}C-methionine (Noltes et al., 2017; Bioletto et al., 2021) and ^{99m}Tc-sestamibi (Whitman et al., 2021; Treglia et al., 2016) were used to assess uptake of the tracer by the PTOs. ^{11}C-methionine is

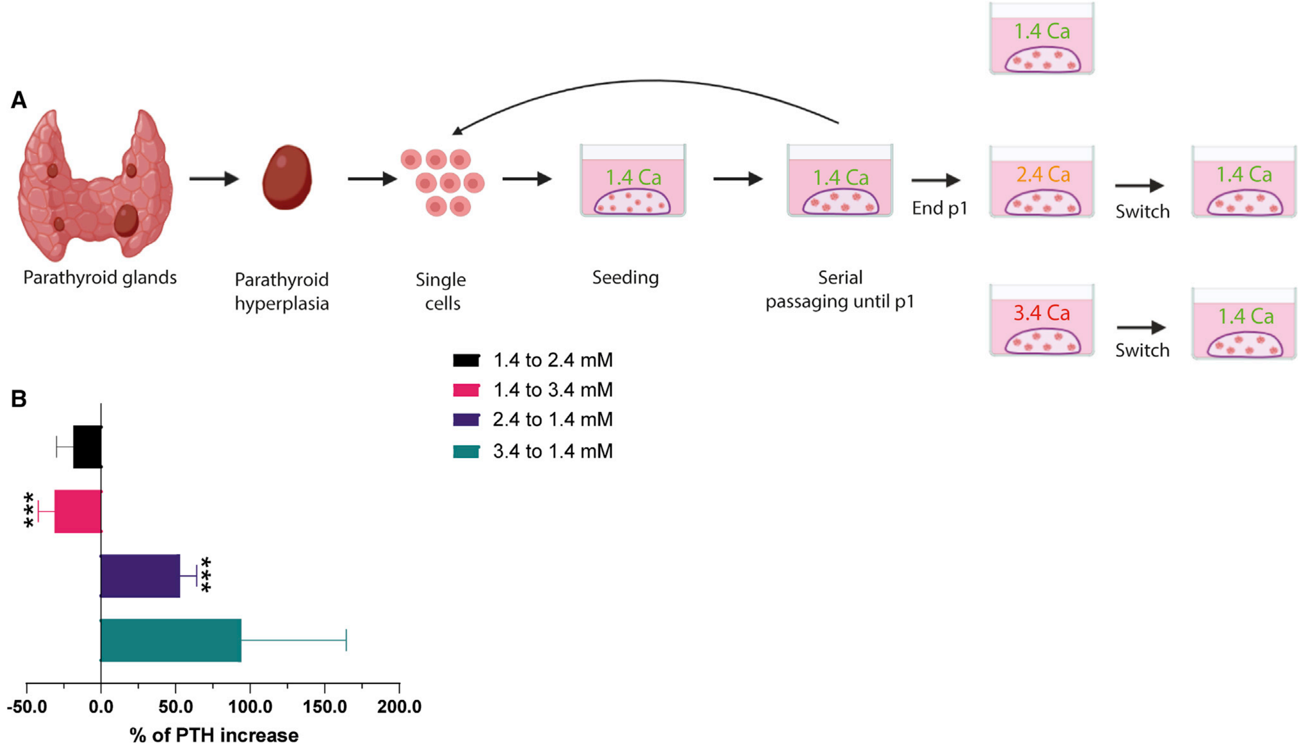

Figure 5. Calcium response experiment of PTOs

(A) Schematic of parathyroid tissue isolation, primary tissue culture, and the calcium-response experiment.

(B) Secreted PTH levels from PTOs in switched calcium concentrations (n = 4 patients from 1.4–2.4 mM, n = 12 patients from 1.4–3.4 mM, n = 4 patients from 2.4–1.4 mM, and n = 4 patients from 3.4–1.4 mM; error bars represent SEM, normalized to PTH levels in the first calcium concentration).

***$p < 0.05$, significant compared with 1.4 mM (baseline).

an amino acid tracer used to localize parathyroid adenomas using a PET scan. The ^{11}C-methionine uptake mechanism is not yet fully understood; it is presumed to be involved in synthesis of pre-pro-PTH, a PTH precursor that results in intense and specific uptake in hyperfunctioning parathyroid glands (Otto et al., 2004). the exact mechanism of ^{99m}Tc-sestamibi uptake in parathyroid glands also remains unknown. ^{99m}Tc-sestamibi uptake depends on numerous factors, including cell cycle phase (Beggs and Hain, 2005). It has been suggested that the electrical potential of the plasma and mitochondrial membrane regulates uptake of ^{99m}Tc-sestamibi (Bandeira et al., 2008; Hetrakul et al., 2001). ^{99m}Tc-sestamibi is detected using a gamma camera, which can acquire 3D SPECT images.

We found that the PTOs showed cell-associated binding of ^{11}C-methionine (cell binding in 10,000 organoids, 0.14% ± 0.09%) and ^{99m}Tc-sestamibi (cell binding in 10,000 organoids, 1.05% ± 0.55%) (Figures 7B and 7D). ^{11}C-methionine showed a significant reduction in cell binding when PTOs were blocked with an excess of L-methionine (0.14% ± 0.09% versus −0.14% ± 0.09%, $p < 0.0005$), indicating specific binding (Figure 7B). When PTOs were incubated with ^{99m}Tc-sestamibi, we observed a higher uptake in PTOs originating from patients with a ^{99m}Tc-sestamibi-positive scan compared with PTOs originating from patients with a ^{99m}Tc-sestamibi-negative scan (1.05% ± 0.55% versus −0.18% ± 0.07%, $p = 0.010$), possibly indicating that we cultured patient-specific PTOs (Figure 7D).

DISCUSSION

In this paper, we report establishment of a patient-derived PTO culture, showing self-renewal potential suggesting the presence of stem cells. We showed that these PTOs resemble the originating tissue on gene and protein expression levels and functionality. We illustrate clinically relevant responses of these PTOs in response to changes in calcium concentration and PTH-lowering drugs. We found specific parathyroid-targeted tracer uptake in our PTOs, demonstrating that PTOs can model human parathyroid functionality.

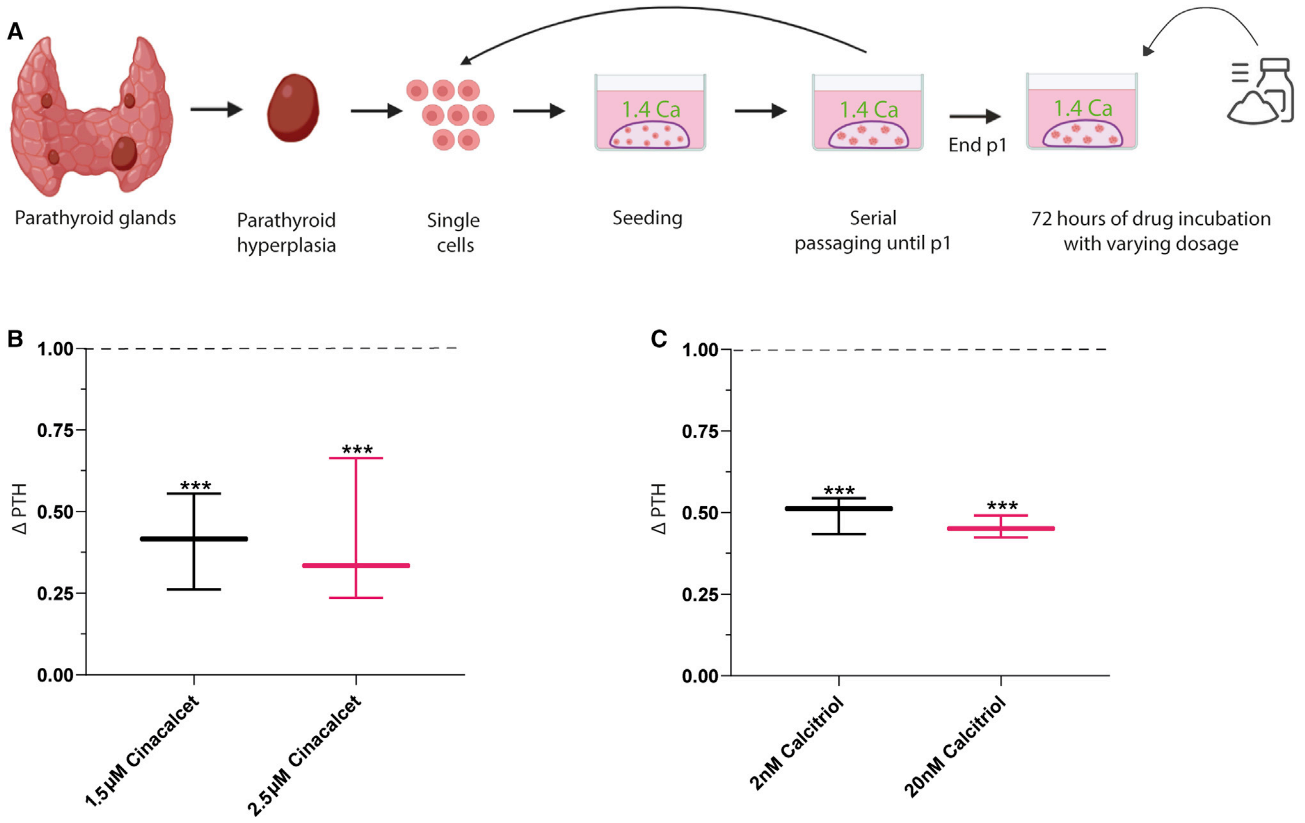

Figure 6. Drug screening experiment of PTOs

(A) Schematic of parathyroid tissue isolation, primary tissue culture, and the drug screening experiment.

(B) Secreted PTH levels from PTOs exposed to 1.5 and 2.5 μM cinacalcet (n = 3 patients; error bars represent SEM, normalized to PTH levels in the DMSO control, dotted line).

(C) Secreted PTH levels from PTOs exposed to 2 and 20 nM calcitriol (n = 3 patients; error bars represent SEM, normalized to PTH levels in the DMSO control, dotted line).

***$p < 0.05$.

Using RNA sequencing, we confirm that our organoids phenocopy parathyroid tissue. In fact, the observed heterogeneity in gene expression between patients seemed to be reflected in the respective organoids. The GO biological process of "regulation of cell population proliferation" was significantly upregulated in dPTOs compared with tissue, in contrast to PTOs, suggesting that this upregulation is the result of exposing PTOs to the modified Bingham differentiation protocol. When organoids were exposed to this differentiation protocol for 2 weeks, it was possible to increase PTH secretion by an additional 50%.

We were unable to find evidence of the existence of unique parathyroid stem cells in our *in vitro* PTO model. This is in line with results from a recent study in thyroid organoids, which could not find evidence of the existence of unique thyroid stem cells (van der Vaart et al., 2021). Many solid tissues do not rely on a professional stem cell for maintenance and repair but temporarily recruit differentiated cells into a transient progenitor pool, suggesting that this phenomenon is also applicable to the thyroid or, in this case, even the parathyroid gland.

For development of new imaging tracers and drugs, an established appropriate pre-clinical model for testing is required (Hicks et al., 2006). Manipulating the calcium concentration and using clinically available PTH-lowering drugs resulted in significantly reduced PTH secretion of PTOs, demonstrating that these organoids can model human parathyroid functionality and are a valid *in vitro* model. This effect is similar to the response expected in patients. We also demonstrated that the PTOs showed specific binding of the PET tracer ^{11}C-methionine and SPECT tracer ^{99m}Tc-sestamibi. This indicates that we may be able to culture patient-specific PTOs. This methodology could also be used to predict patient response to treatment using our organoid model and develop or test new effective parathyroid imaging tracers.

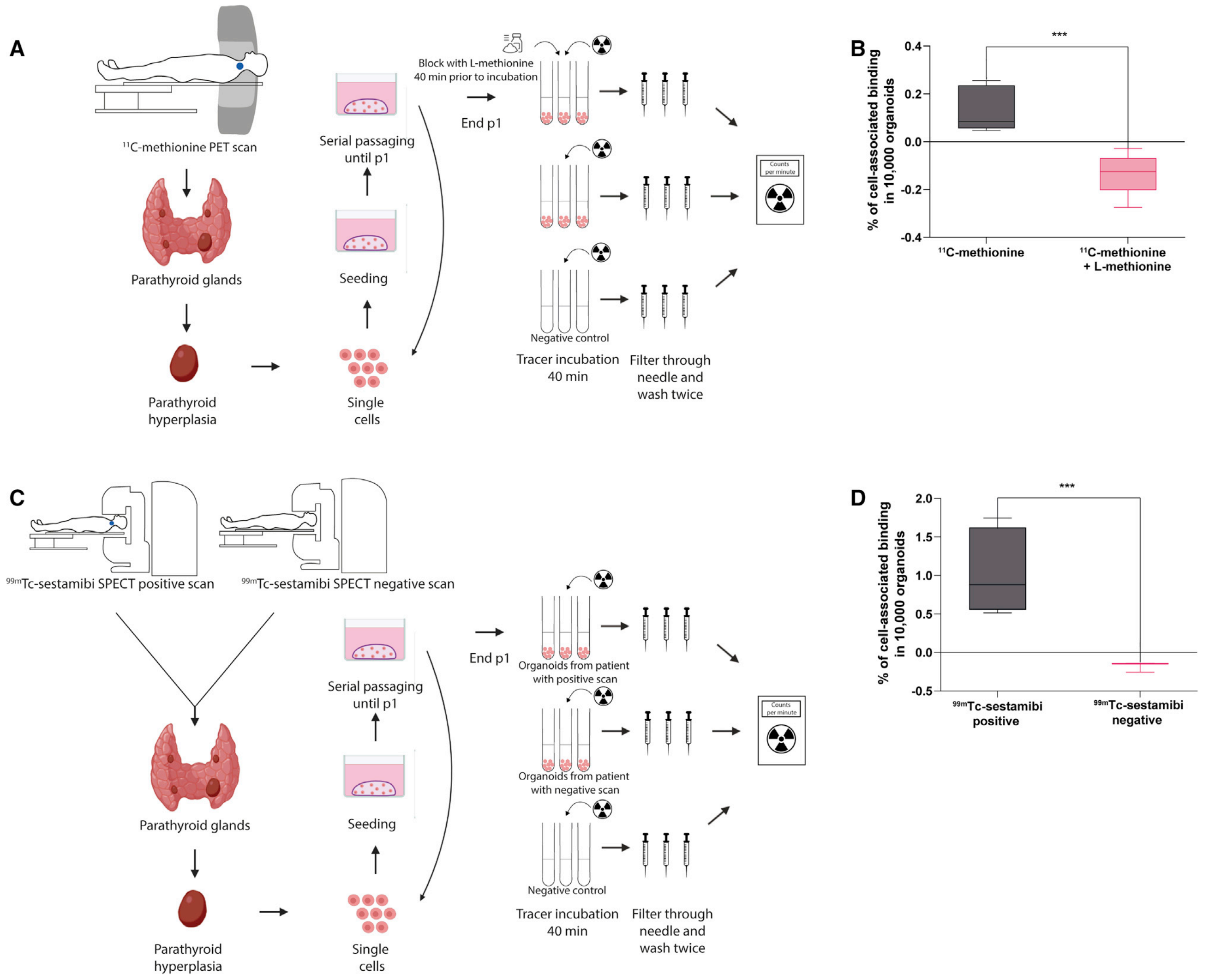

Figure 7. PET/SPECT experiment of PTOs with existing tracers

(A and C) Schematics of parathyroid tissue isolation, primary tissue culture, and the PET/SPECT experiment.

(B) Percentage of ^{11}C-methionine cell-associated binding compared with the percentage of cell-associated binding after blocking with L-methionine (n = 3 patients, Tukey boxplot).

(D) Percentage of ^{99m}Tc-sestamibi cell-associated binding in PTOs originating from patients with a ^{99m}Tc-sestamibi-positive scan (n = 2 patients, Tukey boxplot) compared with the percentage of cell-associated binding in PTOs originating from a patient with a ^{99m}Tc-sestamibi-negative scan (n = 1 patient).

This study is the first to develop a patient-derived PTO culture methodology of parathyroid tissue. Previous studies aimed to culture bovine or rat parathyroid glands in 2D (Fabbri et al., 2014; Brandi et al., 1986; Mithal et al., 1995; Brown et al., 1995). However, those cell culture models of bovine origin failed because of fibroblast cell overgrowth and rapid loss of calcium responsiveness (Brown et al., 1995; Mithal et al., 1995; Ritter et al., 2008). Because cells *in vivo* experience a complex 3D environment that exposes them to circulating molecules, neighboring cells, and the extracellular matrix, 2D cell cultures are unable to mimic the complicated microenvironment cells experience in tissue (Jensen and Teng, 2020). Organoids are self-organizing 3D culture systems that are highly similar to actual human organs, including increased exposure to circulating molecules, neighboring cells, and the extracellular matrix (Matrigel) (Kim et al., 2020; Bayir et al., 2019). Organoids from several glandular tissues have been developed, such as thyroid organoids (Ogundipe et al., 2021), papillary thyroid cancer organoids (Sondorp et al., 2020), salivary glands (Maimets et al., 2016), and prostate cancer organoids (Bahmad

et al., 2018). Analysis of organoid formation could provide valuable insights into the mechanisms underlying human development and organ regeneration (Kim et al., 2020). Such analyses highlight the value of organoids for discovering potential novel therapeutic targets, opening up opportunities for future imaging tracer testing and targeted treatment screening. We encountered a similar effect in our earlier organoid cultures, but lowering the fibroblast growth factor 10 (FGF-10) concentration solved this problem. We did see a small increase in fibroblasts in a later passage (p3). However, these organoids are significantly larger than in earlier passages (p1/p2) and sometimes lack sufficient support from the Matrigel, exposing the PTOs to the topography of the culture plate, potentially leading to epithelial-mesenchymal transition (Hahn et al., 2017).

The limitations of our study include absence of the original microenvironment. This consists of, among others, fluctuating concentrations of extracellular signals and the presence of blood vessels. This may be the reason why our PTOs do not have exactly the same transcriptome as hyperplastic parathyroid tissue. The limited differences that were observed likely do not affect the functionality of our PTOs because the functional testing and tracer experiment show that the PTOs are a highly suitable model that resembles functional parathyroid tissue. It has been shown that microenvironmental factors are able to alter the therapy response of cells, complicating use of organoids for these studies (Ooft et al., 2019; Weeber et al., 2017). Additional research is needed to explore clinical application of this parathyroid culture methodology because one day these PTOs may serve as a stem cell transplantation method for patients with abnormally low levels of PTH (hypoparathyroidism). For this application, first a comparison has to be made between healthy parathyroid tissue and hyperplastic parathyroid tissue.

We present a patient-derived PTO culture that recapitulates the originating tissue on gene and protein expression and functionality. This parathyroid culture will be able to serve as a novel *in vitro* model to perform physiology studies and discover potential novel therapeutic targets, opening opportunities for future tracer testing and drug screening of the parathyroid gland.

EXPERIMENTAL PROCEDURES

Patient material

Human benign hyperplastic parathyroid tissue was obtained from patients undergoing parathyroid surgery. The biopsies were transported from the operating room (OR) in Hank's balanced salt solution (HBSS) (Gibco, NY, USA) with 1% bovine serum albumin (BSA) (Gibco) on ice for immediate processing.

Parathyrosphere cultures

The biopsies were mechanically dispersed using the gentleMACS dissociator (Miltenyi Biotec, Leiden, the Netherlands) and simultaneously subjected to digestion in 5 mL HBSS/1% BSA buffer containing 80 mg/mL dispase (Sigma), 1.2 mg/mL collagenase type II (Gibco), and calcium chloride (Sigma) at a final concentration of 6.25 mM, followed by two periods of 15 min in a 37°C shaking water bath. This isolate was collected by centrifugation, washed in HBSS/1% BSA solution, and passed through a 100-μm cell strainer (BD Biosciences, NJ, USA). The cell pellet was collected by centrifugation and resuspended in Dulbecco's modified Eagle's medium:F12 medium (DMEM:F12) containing Pen/Strep antibiotics (Invitrogen) and Glutamax (Invitrogen). Every 20 μL of this cell solution was combined with 40 μL of Basement Membrane Matrigel (BD Biosciences) on ice and subsequently plated in the center of a 24-well tissue culture plate. After solidification of the gel for 30 min in the incubator, 500 μL of complete growth medium (parathyroid medium) consisting of 50% conditioned Wnt3a medium, 10% conditioned R-Spondin medium, and 40% DMEM:F12 containing B27 (Gibco, 0.5×), HEPES (Gibco, 10 mM), the ROCK inhibitor Y-27632 (10 μM, Abcam, Cambridge, UK), nicotinamide (100 μM, Sigma), FGF-10 (50 ng/mL, PeproTech), Noggin (25 ng/mL, PeproTech, NJ, USA), epidermal growth factor (EGF) (20 ng/mL, Sigma), transforming growth factor β (TGF-β) inhibitor A 83-01 (5 μM, Tocris Bioscience), VEGF-121 (10 ng/mL, Immunotools) and angiotensin (0.1 nmol/mL, Sigma) were added and replaced on a weekly basis.

Self-renewal

To study long-term self-renewing potential, organoids were dissociated and re-plated for the next passage. Prior to passaging, Matrigel was dissolved by incubation with dispase enzyme (1 mg/mL for 30 min at 37°C, Sigma), and organoids with a size greater than 50 μm were counted using an ocular micrometer with a 10× objective. Initial parathyrosphere cultures 7 days of age were dissociated to single cells using 0.05% trypsin-EDTA (Invitrogen) and counted using trypan blue, and cell concentration was adjusted to 2×10^6 cells/mL. In total, 20 μL of this cell solution was combined on ice with 40 μL of Basement Membrane Matrigel (BD Biosciences) and plated in the center of a 24-well tissue culture plate. After solidifying the Matrigel for 30 min at 37°C, gels were covered in 500 μL of parathyroid medium as defined above, and parathyroid medium was renewed weekly. This self-renewal procedure was repeated every 3–4 weeks and up to 5 times (4 passages).

Organoid differentiation

PTOs were encapsulated in Matrigel as described above and subjected to a modified Bingham protocol for differentiation (Bingham et al., 2009; Woods Ignatoski et al., 2010). Standard culture medium, DMEM:F12 containing Pen/Strep antibiotics (Invitrogen), and Glutamax (Invitrogen), was used. In short, cells were continuously exposed to 100 ng/mL Activin A (Immunotools), 100 ng/mL Shh (R&D Systems), and 5% FBS for 14 days with medium changes every 7 days.

PTH secretion

Medium was collected before passage and before each time point during the differentiation period and stored at −20°C until use.

Medium was used to measure the concentration of secreted PTH protein by the PTOs. Secreted PTH was measured by electrochemical luminescence immunoassay (ECLIA) using an Elecsys PTH kit (Roche, Germany).

RNA bulk sequencing

Total RNA was extracted from patient material (n = 3), organoids at the end of p1 (n = 3), and organoids at the end of p1 subjected to 2 weeks of differentiation (n = 3) using the RNeasy Mini Kit (QIAGEN, 74104) according to the manufacturer's protocol. RNA quality (RIN) and quantity were determined using High Sensitivity RNA ScreenTapes (Agilent, 5067). Eight RNA samples with RIN values varying between 7.1 and 9.2 were used for library preparation according to the manufacturer's protocol. For one of the tissue samples, no RNA quality could be detected using the High Sensitivity RNA ScreenTape; therefore, we decided to exclude this sample from further analysis. Quality and concentration of libraries from individual samples were assessed using a fragment analyzer (Agilent). Subsequently, individual libraries were combined into a sequencing pool of 8 samples each with equal molar input. 2 pM was loaded, and 75-bp paired-end sequencing was performed on an Illumina NextSeq 500 system for a 75-bp paired-end sequencing run. For information about qPCR, refer to the supplemental experimental procedures.

RNA sequencing analysis

All bioinformatics analyses were performed with the available packages in RStudio (v.4.0.2), and plots were generated with ggplot2 (v.3.3.3.9000) (Wickham, 2016). Heatmaps were generated with Pheatmap (v.1.0.12) (Kolde, 2019), and row clustering was performed with the ward.D2 clustering method. For the differential gene expression analysis, lowly expressed genes (total count < 1 in fewer than 2 samples) were excluded. The Bioconductor package edgeR (v.3.32.1) (Robinson et al., 2010) was used for normalization by using trimmed mean of M values (TMM) and identification of DEGs (logFC </> 0.95, false discovery rate [FDR] < 0.05) by fitting a generalized linear model. GO analysis for DEGs was performed with enrichR (v.3.0) (Xie et al., 2021). The database GO_Biological_Process_2021 was used to identify enriched GO terms.

Immunostaining

For immunostaining, antibodies to PTH (1:100, AB_2924262), CaSR (1:100, AB_444867), GCM2 (1:100, AB_10733096), and CHGA (1:100, AB_2918358) were used to detect proteins. In the case of the organoids, Matrigel was dissolved by incubation with dispase enzyme, followed by washes with PBS/0.2% BSA and centrifuged at 400 × *g* for 5 min. The resulting pellet was fixed in 4% paraformaldehyde (15 min, room temperature [RT]) and washed with PBS. Next, the organoids were embedded in HistoGel (Richard-Allan Scientific/Thermo Scientific), and the gel was subjected to dehydration, followed by embedding in paraffin and sectioning (5 μm). Patient material was fixed in 4% formaldehyde. After dehydration, the tissue was paraffin embedded and sectioned at 5-μm thickness. Sections were de-paraffinized and Tris-EDTA antigen retrieval was performed. This was followed by washing, blocking, and incubation with primary antibodies overnight. Slides were then incubated with secondary antibodies, stained with 4′,6-diamidino-2-phenylindole (DAPI), and mounted with aqueous mounting medium (Dako) for tissue sections or Prolong Glass Antifade Mountant (Thermo Fisher Scientific) for organoid sections. Imaging was performed using a Leica TCS SP8 X confocal microscope.

Effect of extracellular calcium on PTH secretion

Undifferentiated organoids were exposed to parathyroid medium containing a normal (1.4 mM), high (2.4 mM), or higher (3.4 mM) concentration of calcium for 7 days. Medium calcium content was measured by photometrical absorption measurement of calcium EDTA complex using the Calcium Gen.2 kit (Roche, Germany). After 7 days, the medium was collected to measure the secreted concentration of PTH. Then organoids were switched from a normal (1.4 mM) to a higher (3.4 mM) concentration of calcium or vice versa to study their rescue potential. After 7 days, the medium was again collected to measure the secreted concentration of PTH.

Drug screening

Cinacalcet (Combi-Blocks, QA-8513) was added to the medium at two different concentrations (1.5 and 2.5 μM), and after 72 h of incubation, the medium was collected to measure the concentration of secreted PTH protein by the PTOs. The concentration of cinacalcet used was based on studies that investigated the effect on parathyroid cultured cells (Tatsumi et al., 2013; Kawata et al., 2006). Calcitriol (Cayman Chemical, 32222-06-3) was added to the medium at two concentrations (2 and 20 nM), and after 72 h of incubation, the medium was collected to measure the concentration of secreted PTH protein by PTOs. The concentration of calcitriol used was also based on a previous study (Canalejo et al., 2000). Cinacalcet and calcitriol were dissolved using DMSO; therefore, DMSO was used as a vehicle control at a concentration of 0.1%.

PET/SPECT tracer validation

To demonstrate the utility of patient-derived PTOs to validate PET/SPECT tracers, existing tracers, ^{11}C-methionine and ^{99m}Tc-sestamibi, were used to assess uptake of the tracer by the PTOs.

Prior to the experiments, Matrigel was dissolved by incubation with dispase enzyme, followed by three washes with 1,000 μL of HBSS/1% BSA. Approximately 10,000 organoids in 450 μL HBSS/1% BSA were incubated with 2 MBq (in 50 μL) tracer solution at 37°C for 40 min. For ^{11}C-mehionine, organoids were blocked with 2 μM (in 50 μL) of cold L-methionine (Sigma-Aldrich) 40 min prior to tracer incubation. After tracer incubation, the organoids were collected with a 5-μm filter needle and washed twice through the filter needle with 500 μL ice-cold HBSS/1% BSA. Activity in the organoids was measured in a gamma counter (Wizard^2 2480, SW 2.1, PerkinElmer). Tracer uptake was corrected for the number of organoids by counting the number of organoids using an ocular micrometer with a 10× objective prior to the experiment. Uptake was expressed as a percentage of organoid-associated radioactivity per 10,000 organoids. Uptake values were corrected for background radiation. All experiments were executed in duplicate or triplicate, depending on the number of organoids.

Statistics

All data represent the mean ± SEM. Statistical analyses and production of graphs were done using GraphPad Prism v.8 (GraphPad). The statistical methods relevant to each figure are described in the figure legends. Statistical comparisons between two groups were performed using Mann-Whitney U test or Student's t test (depending on normal distribution). p values of less than 0.05 were considered to indicate a significant difference between groups.

Study approval

Written informed consent of participants was obtained prior to participation. The study was approved by the Medical Ethics Committee Groningen (approval no. 2017/640).

DATA AND CODE AVAILABILITY

The raw bulk RNA sequencing data (fastq files) used in this study have been deposited in the NCBI's Gene Expression Omnibus (GEO) (GEO: GSE197445).

SUPPLEMENTAL INFORMATION

Supplemental information can be found online at https://doi.org/10.1016/j.stemcr.2022.09.015.

AUTHOR CONTRIBUTIONS

Conceptualization, M.E.N., L.H.J.S., R.P.C., and S.K.; methodology, M.E.N. and L.H.J.S.; formal analysis, M.E.N., L.H.J.S., L.K., I.F.A., and R.W.; investigation, M.E.N., I.F.A., and L.H.J.S.; resources, R.P.C., I.F.A., W.S., A.H.B., W.K., L.J., W.T.Z., A.K., and S.K.; data curation, M.E.N., L.H.J.S., R.P.C., and S.K.; writing – original draft, M.E.N. and L.H.J.S.; writing – review & editing, M.E.N., L.H.J.S., L.K., I.F.A., R.W., W.K., A.K., W.S., W.T.Z., L.J., A.H.B., R.P.C., and S.K.; visualization, M.E.N. and L.H.J.S.; supervision, R.P.C., A.H.B., and S.K.; project administration, R.P.C. and S.K.; funding acquisition, R.P.C. and S.K.

ACKNOWLEDGMENTS

We thank the surgeons and pathology departments of the University Medical Center Groningen, Treant location Bethesda, and the Martini Hospital for collecting and distributing hyperplastic parathyroid gland tissue. Sequencing was performed with excellent assistance from Diana Spierings and the ERIBA Research Sequencing Facility. Part of the work was performed at the UMCG Imaging and Microscopy Center (UMIC). This work was partly funded by UMCG Cancer Research Fund 490178 (to R.P.C. and S.K.).

CONFLICT OF INTERESTS

The authors declare no competing interests.

Received: June 13, 2022
Revised: September 29, 2022
Accepted: September 29, 2022
Published: October 27, 2022

REFERENCES

Bahmad, H.F., Cheaito, K., Chalhoub, R.M., Hadadeh, O., Monzer, A., Ballout, F., El-Hajj, A., Mukherji, D., Liu, Y.N., Daoud, G., and Abou-Kheir, W. (2018). Sphere-formation assay: three-dimensional in vitro culturing of prostate cancer stem/progenitor sphere-forming cells. Front. Oncol. *8*, 347. https://doi.org/10.3389/fonc.2018.00347.

Bandeira, F.A.F., Oliveira, R.I.R.B., Griz, L.H.M., Caldas, G., and Bandeira, C. (2008). Differences in accuracy of 99mTc-sestamibi scanning between severe and mild forms of primary hyperparathyroidism. J. Nucl. Med. Technol. *36*, 30–35. https://doi.org/10.2967/jnmt.107.044040.

Bayir, E., Sendemir, A., and Missirlis, Y.F. (2019). Mechanobiology of cells and cell systems, such as organoids. Biophys. Rev. *11*, 721–728. https://doi.org/10.1007/s12551-019-00590-7.

Beggs, A.D., and Hain, S.F. (2005). Localization of parathyroid adenomas using 11C-methionine positron emission tomography. Nucl. Med. Commun. *26*, 133–136.

Bingham, E.L., Cheng, S.P., Woods Ignatoski, K.M., and Doherty, G.M. (2009). Differentiation of human embryonic stem cells to a parathyroid-like phenotype. Stem Cell. Dev. *18*, 1071–1080. https://doi.org/10.1089/scd.2008.0337.

Bioletto, F., Barale, M., Parasiliti-Caprino, M., Prencipe, N., Berton, A.M., Procopio, M., Deandreis, D., and Ghigo, E. (2021). Comparison of the diagnostic accuracy of 18F-Fluorocholine PET and 11C-Methionine PET for parathyroid localization in primary hyperparathyroidism: a systematic review and meta-analysis. Eur. J. Endocrinol. *185*, 109–120.

Brandi, M.L., Fitzpatrick, L.A., Coon, H.G., and Aurbach, G.D. (1986). Bovine parathyroid cells: cultures maintained for more than 140 population doublings. Proc. Natl. Acad. Sci. USA *83*, 1709–1713. https://doi.org/10.1073/pnas.83.6.1709.

Brown, A.J., Zhong, M., Ritter, C., Brown, E.M., and Slatopolsky, E. (1995). Loss of calcium responsiveness in cultured bovine parathyroid cells is associated with decreased calcium receptor expression. Biochem. Biophys. Res. Commun. *212*, 861–867.

Canalejo, A., Almadén, Y., Torregrosa, V., Gomez-Villamandos, J.C., Ramos, B., Campistol, J.M., Felsenfeld, A.J., and Rodríguez, M. (2000). The in vitro effect of calcitriol on parathyroid cell proliferation and apoptosis. J. Am. Soc. Nephrol. *11*, 1865–1872. https://doi.org/10.1681/ASN.V11101865.

Christie, A.C. (1967). The parathyroid oxyphil cells. J. Clin. Pathol. *20*, 591–602. https://doi.org/10.1136/jcp.20.4.591.

Fabbri, S., Ciuffi, S., Nardone, V., Gomes, A.R., Mavilia, C., Zonefrati, R., Galli, G., Luzi, E., Tanini, A., and Brandi, M.L. (2014). PTH-C1: a rat continuous cell line expressing the parathyroid phenotype. Endocrine *47*, 90–99. https://doi.org/10.1007/s12020-014-0229-7.

Fagerberg, L., Hallström, B.M., Oksvold, P., Kampf, C., Djureinovic, D., Odeberg, J., Habuka, M., Tahmasebpoor, S., Danielsson, A., Edlund, K., et al. (2014). Analysis of the human tissue-specific expression by genome-wide integration of transcriptomics and antibody-based proteomics. Mol. Cell. Proteomics *13*, 397–406. https://doi.org/10.1074/mcp.M113.035600.

Gendoo, D.M.A., Denroche, R.E., Zhang, A., Radulovich, N., Jang, G.H., Lemire, M., Fischer, S., Chadwick, D., Lungu, I.M.,

Ibrahimov, E., et al. (2019). Whole genomes define concordance of matched primary, xenograft, and organoid models of pancreas cancer. PLoS Comput. Biol. *15*, e1006596. https://doi.org/10.1371/journal.pcbi.1006596.

Hahn, S., Nam, M.O., Noh, J.H., Lee, D.H., Han, H.W., Kim, D.H., Hahm, K.B., Hong, S.P., Yoo, J.H., and Yoo, J. (2017). Organoid-based epithelial to mesenchymal transition (OEMT) model: from an intestinal fibrosis perspective. Sci. Rep. *7*, 2435. https://doi.org/10.1038/s41598-017-02190-5.

Hetrakul, N., Civelek, A.C., Stagg, C.A., and Udelsman, R. (2001). In vitro accumulation of technetium-99m-sestamibi in human parathyroid mitochondria. Surgery *130*, 1011–1018.

Hicks, R.J., Dorow, D., and Roselt, P. (2006). PET tracer development–a tale of mice and men. Cancer Imag. *6*, 102–S106. https://doi.org/10.1102/1470-7330.2006.9098.

Hinson, J., Raven, P., and Chew, S. (2010). 12 - hormonal regulation of plasma calcium and calcium metabolism. In The Endocrine System, Second Edition, J. Hinson, P. Raven, and S. Chew, eds. (Churchill Livingstone), pp. 147–159.

Hu, H., Gehart, H., Artegiani, B., LÖpez-Iglesias, C., Dekkers, F., Basak, O., van Es, J., Chuva de Sousa Lopes, S.M., Begthel, H., Korving, J., et al. (2018). Long-term expansion of functional mouse and human hepatocytes as 3D organoids. Cell *175*, 1591–1606.e19.

Jensen, C., and Teng, Y. (2020). Is it time to start transitioning from 2D to 3D cell culture? Front. Mol. Biosci. *7*, 33. https://doi.org/10.3389/fmolb.2020.00033.

Junaid, Z., and Patel, J. (2021). Cinacalcet, 2021.

Kawata, T., Imanishi, Y., Kobayashi, K., Onoda, N., Okuno, S., Takemoto, Y., Komo, T., Tahara, H., Wada, M., Nagano, N., et al. (2006). Direct in vitro evidence of the suppressive effect of cinacalcet HCl on parathyroid hormone secretion in human parathyroid cells with pathologically reduced calcium-sensing receptor levels. J. Bone Miner. Metabol. *24*, 300–306. https://doi.org/10.1007/s00774-006-0687-y.

Kim, J., Koo, B.K., and Knoblich, J.A. (2020). Human organoids: model systems for human biology and medicine. Nat. Rev. Mol. Cell Biol. *21*, 571–584. https://doi.org/10.1038/s41580-020-0259-3.

Kolde, R. (2019). pheatmap: Pretty Heatmaps. 1.0.12.

Maimets, M., Rocchi, C., Bron, R., Pringle, S., Kuipers, J., Giepmans, B.N.G., Vries, R.G.J., Clevers, H., de Haan, G., van Os, R., and Coppes, R.P. (2016). Long-term in vitro expansion of salivary gland stem cells driven by Wnt signals. Stem Cell Rep. *6*, 150–162.

Mannstadt, M. (2021). Parathyroid Hormone Secretion and Action.

McCracken, K.W., Catá, E.M., Crawford, C.M., Sinagoga, K.L., Schumacher, M., Rockich, B.E., Tsai, Y.H., Mayhew, C.N., Spence, J.R., Zavros, Y., and Wells, J.M. (2014). Modelling human development and disease in pluripotent stem-cell-derived gastric organoids. Nature *516*, 400–404. https://doi.org/10.1038/nature13863.

Mithal, A., Kifor, O., Kifor, I., Vassilev, P., Butters, R., Krapcho, K., Simin, R., Fuller, F., Hebert, S.C., and Brown, E.M. (1995). The reduced responsiveness of cultured bovine parathyroid cells to extracellular Ca2+ is associated with marked reduction in the expression of extracellular Ca(2+)-sensing receptor messenger ribonucleic acid and protein. Endocrinology *136*, 3087–3092. https://doi.org/10.1210/endo.136.7.7789335.

Noltes, M.E., Coester, A.M., van der Horst-Schrivers, A.N.A., Dorgelo, B., Jansen, L., Noordzij, W., Lemstra, C., Brouwers, A.H., and Kruijff, S. (2017). Localization of parathyroid adenomas using 11C-methionine pet after prior inconclusive imaging. Langenbecks Arch. Surg. *402*, 1109–1117.

Ogundipe, V.M.L., Groen, A.H., Hosper, N., Nagle, P.W.K., Hess, J., Faber, H., Jellema, A.L., Baanstra, M., Links, T.P., Unger, K., et al. (2021). Generation and differentiation of adult tissue-derived human thyroid organoids. Stem Cell Rep. *16*, 913–925.

Ooft, S.N., Weeber, F., Dijkstra, K.K., McLean, C.M., Kaing, S., van Werkhoven, E., Schipper, L., Hoes, L., Vis, D.J., van de Haar, J., et al. (2019). Patient-derived organoids can predict response to chemotherapy in metastatic colorectal cancer patients. Sci. Transl. Med. *11*, eaay2574. https://doi.org/10.1126/scitranslmed.aay2574.

Ordóñez, N.G. (2014). Value of GATA3 immunostaining in the diagnosis of parathyroid tumors. Appl. Immunohistochem. Mol. Morphol. *22*, 756–761. https://doi.org/10.1097/PAI.0000000000000007.

Otto, D., Boerner, A.R., Hofmann, M., Brunkhorst, T., Meyer, G.J., Petrich, T., Scheumann, G.F., and Knapp, W.H. (2004). Pre-operative localisation of hyperfunctional parathyroid tissue with 11C-methionine PET. Eur. J. Nucl. Med. Mol. Imag. *31*, 1405–1412. https://doi.org/10.1007/s00259-004-1610-1.

Park, Y.S., Kim, H.S., Jin, Y.M., Yu, Y., Kim, H.Y., Park, H.S., Jung, S.C., Han, K.H., Park, Y.J., Ryu, K.H., and Jo, I. (2015). Differentiated tonsil-derived mesenchymal stem cells embedded in Matrigel restore parathyroid cell functions in rats with parathyroidectomy. Biomaterials *65*, 140–152. https://doi.org/10.1016/j.biomaterials.2015.06.044.

Pringle, S., Maimets, M., van der Zwaag, M., Stokman, M.A., van Gosliga, D., Zwart, E., Witjes, M.J.H., de Haan, G., van Os, R., and Coppes, R.P. (2016). Human salivary gland stem cells functionally restore radiation damaged salivary glands. Stem Cell. *34*, 640–652. https://doi.org/10.1002/stem.2278.

Ritter, C.S., Haughey, B.H., Miller, B., and Brown, A.J. (2012). Differential gene expression by oxyphil and chief cells of human parathyroid glands. J. Clin. Endocrinol. Metab. *97*, 1499–E1505. https://doi.org/10.1210/jc.2011-3366.

Ritter, C.S., Pande, S., Krits, I., Slatopolsky, E., and Brown, A.J. (2008). Destabilization of parathyroid hormone mRNA by extracellular Ca2+ and the calcimimetic R-568 in parathyroid cells: role of cytosolic Ca and requirement for gene transcription. J. Mol. Endocrinol. *40*, 13–21.

Robinson, M.D., McCarthy, D.J., and Smyth, G.K. (2010). edgeR: a Bioconductor package for differential expression analysis of digital gene expression data. Bioinformatics *26*, 139–140. https://doi.org/10.1093/bioinformatics/btp616.

Singh, P., Bhadada, S.K., Dahiya, D., Arya, A.K., Saikia, U.N., Sachdeva, N., Kaur, J., Brandi, M.L., and Rao, S.D. (2020). Reduced calcium sensing receptor (CaSR) expression is epigenetically deregulated in parathyroid adenomas. J. Clin. Endocrinol. Metab. *105*. dgaa419–3024.

Sondorp, L.H.J., Ogundipe, V.M.L., Groen, A.H., Kelder, W., Kemper, A., Links, T.P., Coppes, R.P., and Kruijff, S. (2020). Patient-derived papillary thyroid cancer organoids for radioactive iodine

refractory screening. Cancers *12*, 3212. https://doi.org/10.3390/cancers12113212.

Tatsumi, R., Komaba, H., Kanai, G., Miyakogawa, T., Sawada, K., Kakuta, T., and Fukagawa, M. (2013). Cinacalcet induces apoptosis in parathyroid cells in patients with secondary hyperparathyroidism: histological and cytological analyses. Nephron Clin. Pract. *124*, 224–231. https://doi.org/10.1159/000357951.

Tfelt-Hansen, J., and Brown, E.M. (2005). The calcium-sensing receptor in normal physiology and pathophysiology: a review. Crit. Rev. Clin. Lab Sci. *42*, 35–70. https://doi.org/10.1080/10408360590886606.

The Human Protein Atlas (2022). The parathyroid Gland-specific proteome.

Treglia, G., Sadeghi, R., Schalin-Jäntti, C., Caldarella, C., Ceriani, L., Giovanella, L., and Eisele, D.W. (2016). Detection rate of (99m) Tc-MIBI single photon emission computed tomography (SPECT)/CT in preoperative planning for patients with primary hyperparathyroidism: a meta-analysis. Head Neck *38* (*Suppl 1*), 2159–E2172. https://doi.org/10.1002/hed.24027.

Tuveson, D., and Clevers, H. (2019). Cancer modeling meets human organoid technology. Science *364*, 952–955. https://doi.org/10.1126/science.aaw6985.

Uhlén, M., Fagerberg, L., Hallström, B.M., Lindskog, C., Oksvold, P., Mardinoglu, A., Sivertsson, Å., Kampf, C., Sjöstedt, E., Asplund, A., et al. (2015). Proteomics. Tissue-based map of the human proteome. Science *347*, 1260419. https://doi.org/10.1126/science.1260419.

van der Vaart, J., Bosmans, L., Sijbesma, S.F., Knoops, K., van de Wetering, W.J., Otten, H.G., Begthel, H., Borel Rinkes, I.H.M., Korving, J., Lentjes, E.G.W.M., et al. (2021). Adult mouse and human organoids derived from thyroid follicular cells and modeling of Graves' hyperthyroidism. Proc. Natl. Acad. Sci. USA *118*. e2117017118. https://doi.org/10.1073/pnas.2117017118.

Varshney, S., Bhadada, S.K., Saikia, U.N., Sachdeva, N., Behera, A., Arya, A.K., Sharma, S., Bhansali, A., Mithal, A., and Rao, S.D. (2013). Simultaneous expression analysis of vitamin D receptor, calcium-sensing receptor, cyclin D1, and PTH in symptomatic primary hyperparathyroidism in Asian Indians. Eur. J. Endocrinol. *169*, 109–116. https://doi.org/10.1530/EJE-13-0085.

Weeber, F., Ooft, S.N., Dijkstra, K.K., and Voest, E.E. (2017). Tumor organoids as a pre-clinical cancer model for drug discovery. Cell Chem. Biol. *24*, 1092–1100.

Whitman, J., Allen, I.E., Bergsland, E.K., Suh, I., and Hope, T.A. (2021). Assessment and comparison of (18)F-fluorocholine PET and (99m)Tc-sestamibi scans in identifying parathyroid adenomas: a metaanalysis. J. Nucl. Med. *62*, 1285–1291. https://doi.org/10.2967/jnumed.120.257303.

Wickham, H. (2016). ggplot2: Elegant Graphics for Data Analysis (Springer).

Woods Ignatoski, K.M., Bingham, E.L., Frome, L.K., and Doherty, G.M. (2010). Differentiation of precursors into parathyroid-like cells for treatment of hypoparathyroidism. Surgery *148*, 1186–1189. discussion 1189-1190. https://doi.org/10.1016/j.surg.2010.09.021.

Xie, Z., Bailey, A., Kuleshov, M.V., Clarke, D.J.B., Evangelista, J.E., Jenkins, S.L., Lachmann, A., Wojciechowicz, M.L., Kropiwnicki, E., Jagodnik, K.M., et al. (2021). Gene set knowledge discovery with enrichr. Curr. Protoc. *1*, e90. https://doi.org/10.1002/cpz1.90.

Yu, N.Y.L., Hallström, B.M., Fagerberg, L., Ponten, F., Kawaji, H., Carninci, P., Forrest, A.R.R., Fantom Consortium, Hayashizaki, Y., Uhlén, M., and Daub, C.O. (2015). Complementing tissue characterization by integrating transcriptome profiling from the Human Protein Atlas and from the FANTOM5 consortium. Nucleic Acids Res. *43*, 6787–6798. https://doi.org/10.1093/nar/gkv608.

Stem Cell Reports, Volume *17*

Supplemental Information

Patient-derived parathyroid organoids as a tracer and drug-screening application model

Milou E. Noltes, Luc H.J. Sondorp, Laura Kracht, Inês F. Antunes, René Wardenaar, Wendy Kelder, Annelies Kemper, Wiktor Szymanski, Wouter T. Zandee, Liesbeth Jansen, Adrienne H. Brouwers, Robert P. Coppes, and Schelto Kruijff

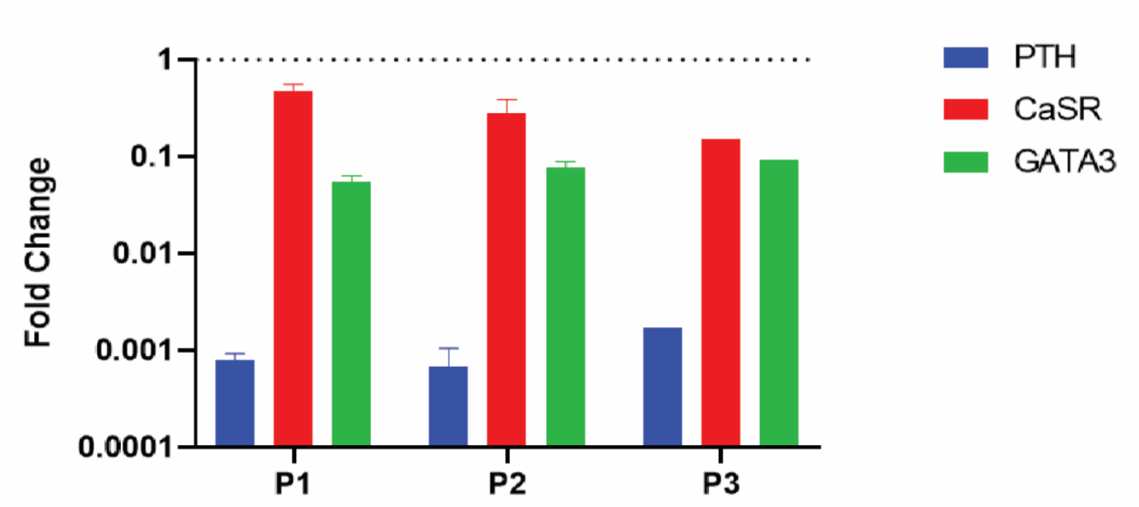

Supplemental Figure 1. Gene expression pattern analysis across multiple passages (n=3 patients for p1 and p2, n=2 for p3). Dotted line resembles tissue expression levels (n=3), and error bars resemble SEM. No significance was observed between passages with the lowest p-value being 0.065. Related to Figure 1 (self-renewal of organoid-forming cells from human putative parathyroid stem cells).

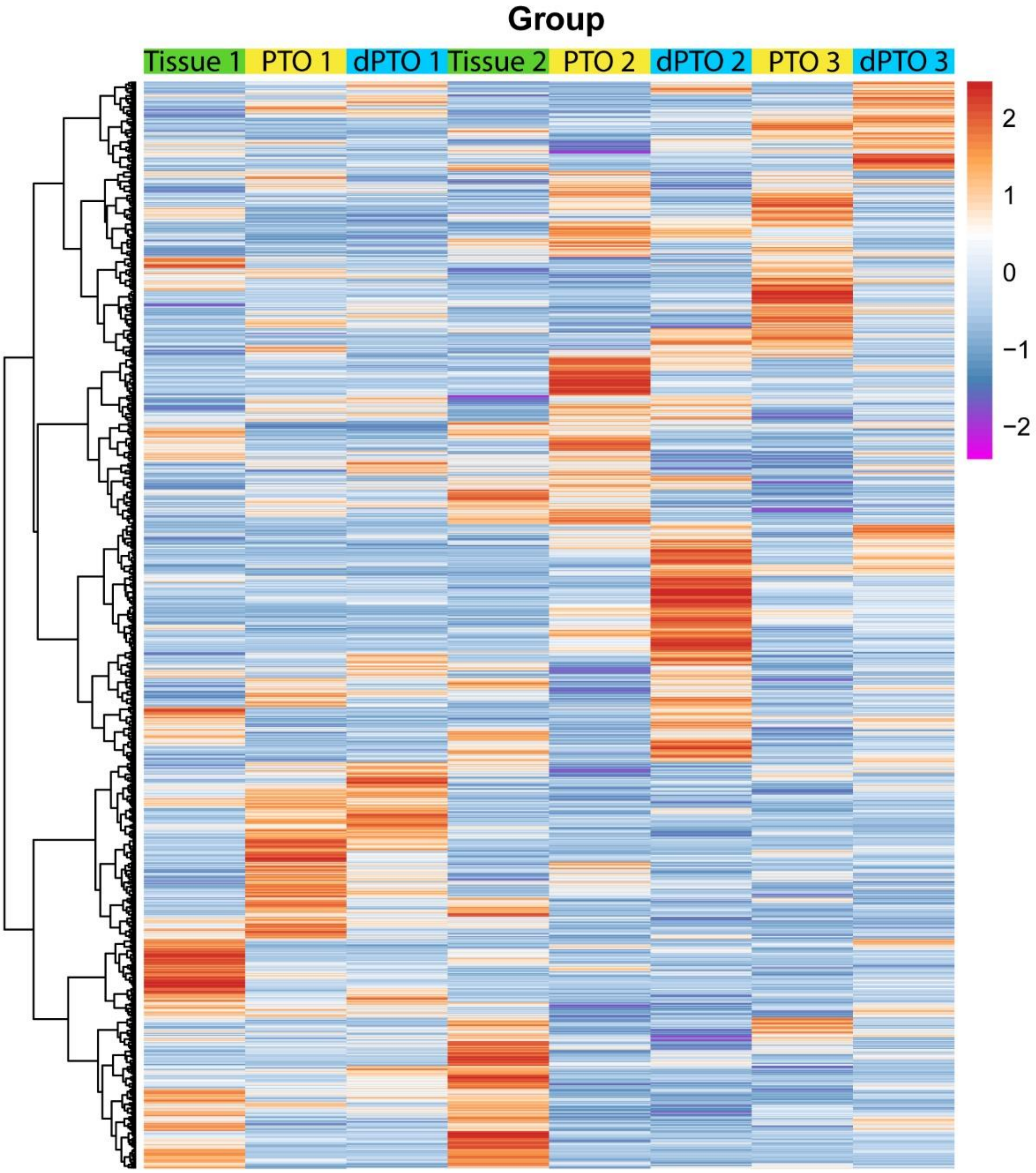

Supplemental Figure 2. Heatmap showing all sequenced genes for all included samples separately. Data is shown in row Z-score of counts per million (Supplemental table 1). *PTO*= parathyroid organoids, *dPTO*= differentiated parathyroid organoids. Related to Figure 3 (bulk RNA-sequencing).

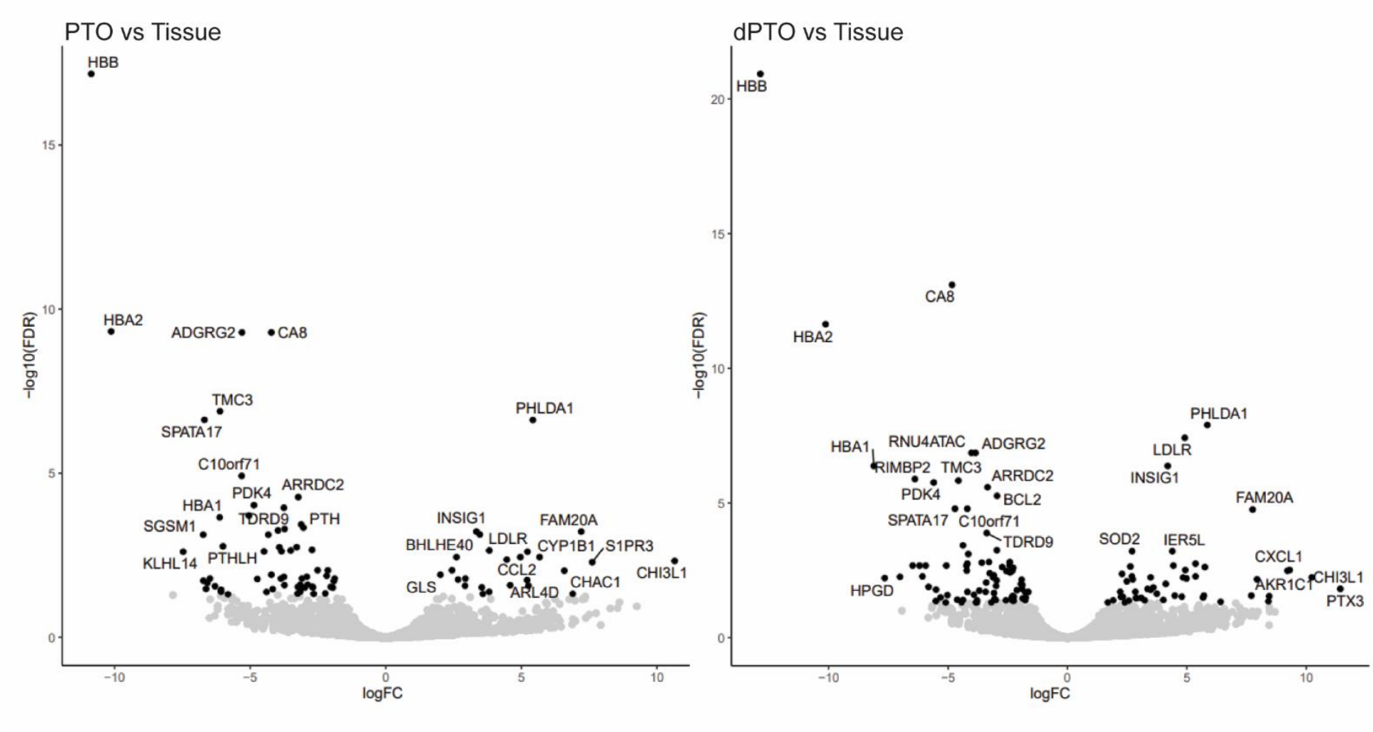

Supplemental Figure 3. Volcano plots showing differentially expressed genes (FDR<0.05) in PTO or dPTO compared to tissue (Supplemental Table 2 and 3). *PTO*= parathyroid organoids, *dPTO*= differentiated parathyroid organoids. Related to Figure 3 (bulk RNA-sequencing).

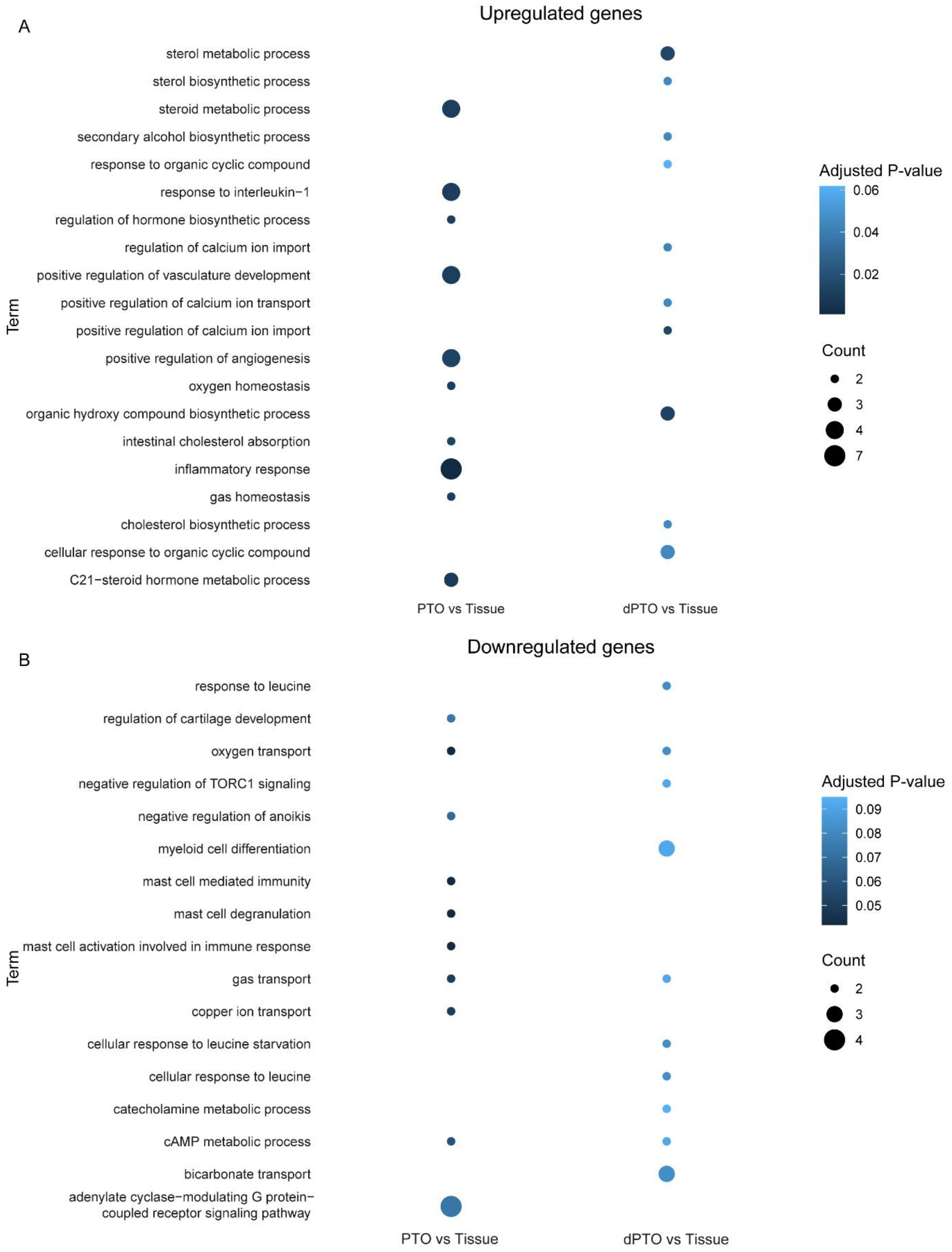

Supplemental Figure 4. Dotplots showing top ten GO terms associated with upregulated (A) and downregulated (B) genes in PTO and dPTO compared to tissue. Ranking of GO terms was based on the most significant GO terms. Dot colors resemble significance and size resembles number of associated genes *PTO*= parathyroid organoids, *dPTO*= differentiated parathyroid organoids. Related to Figure 3 (bulk RNA-sequencing).

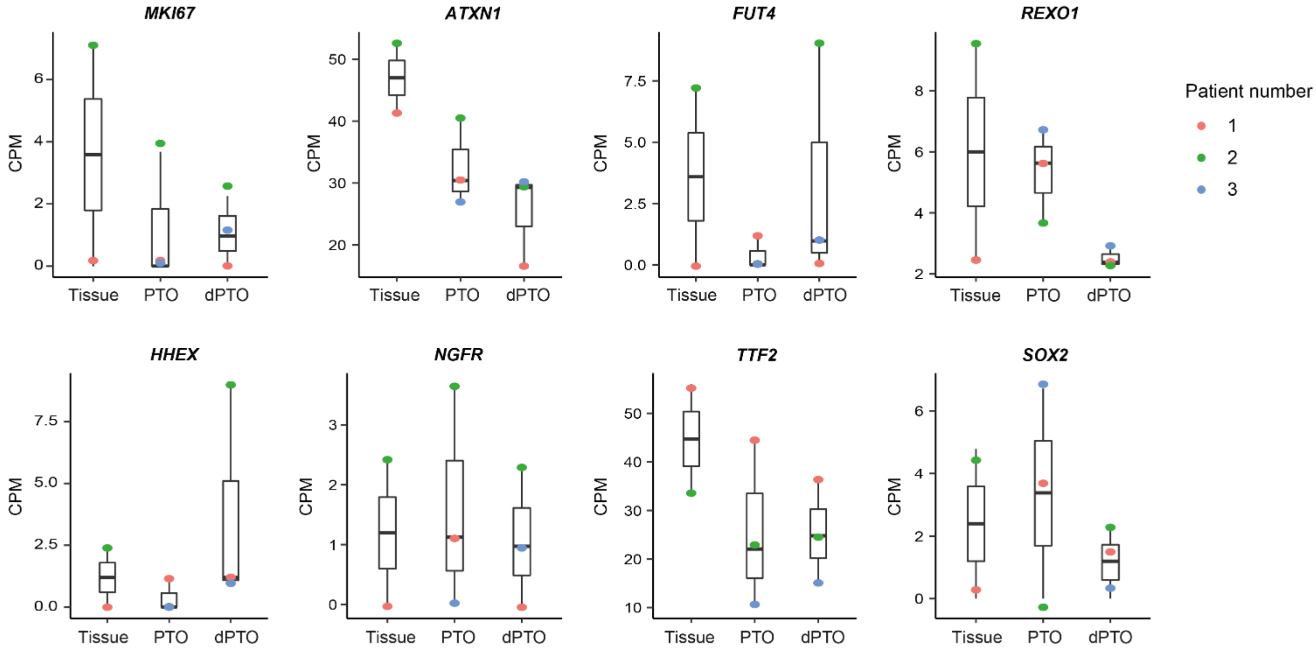

Supplemental Figure 5. Boxplots of generic stem cell markers of primary hyperplastic parathyroid tissue, PTOs at the end of passage 1 and two weeks dPTOs in counts per million (CPM). Boxplot shows median, two hinges (25th and 75th percentile) and two whiskers (largest and smallest value no further than 1.5x inter-quartile range). *PTO*= parathyroid organoids, *dPTO*= differentiated parathyroid organoids. Related to Figure 3 (bulk RNA-sequencing).

Supplemental experimental procedures

Quantitative Polymerase Chain Reaction

Total RNA from patient material (n=3) and organoids (p1 and p2 n=3, p3 n=2) was extracted (RNeasy™ Mini Kit ,Qiagen). To obtain cDNA, 500ng of total RNA was reverse transcribed using 1 µL 10 mM dNTP Mix, 100 ng random primers, 5x First-strand Buffer, 0.1 M DTT, 40 units of RNase OUT and 200 units of M-MLV RT, in a volume of 20 µL for each reaction (all Invitrogen). qPCR (Bio-Rad) was performed using Bio-Rad iQ SYBR Green Supermix according to manufacturer's instructions. For each sample, PCR buffer was mixed with 100 ng cDNA, sybergreen and forward and reverse primers for the targeted genes in a volume of 13 µL. A three-step qPCR reaction was applied. Oligo sequences of primes were as followed: PTH fwd, 5′-AGCTACTAACATACCTGAACG-3′; PTH rev, 5′-CTCTCCATCGACTTCAGATG-3′; CaSR fwd, 5′-AGATGGCACGGGACACTACC-3′; CaSR rev, 5′-AGGAGGCATAACTGACCTGGG-3′; GATA3 fwd, 5′-AAGCCTCTGCAATGTGCTC-3′; GATA3 rev, 5′-GTGGTGGTCTGACAGTTCGC-3′, and YWHAZ fwd, 5′- GATCCCCAATGCTTCACAAG-3′; YWHAZ rev, 5′- TGCTTGTTGTGACTGATCGAC -3′.
